# Synthetic approaches to bowl-shaped π-conjugated sumanene and its congeners

**DOI:** 10.3762/bjoc.16.186

**Published:** 2020-09-09

**Authors:** Shakeel Alvi, Rashid Ali

**Affiliations:** 1 Department of Chemistry, Jamia Millia Islamia, Jamia Nagar, Okhla, New Delhi-110025, India, Phone: +91-7011867613

**Keywords:** buckybowls, heterosumanenes, polyaromatic hydrocarbons, sumanene, synthesis

## Abstract

Since the first synthetic report in 2003 by Sakurai et al., sumanene (derived from the Indian ‘Hindi as well as Sanskrit word’ “Suman”, which means “Sunflower”), a beautifully simple yet much effective bowl-shaped *C*_3_-symmetric polycyclic aromatic hydrocarbon having three benzylic positions clipped between three phenyl rings in the triphenylene framework has attracted a tremendous attention of researchers worldwide. Therefore, since its first successful synthesis, a variety of functionalized sumanenes as well as heterosumanenes have been developed because of their unique physiochemical properties. For example, bowl-to-bowl inversion, bowl depth, facial selectivity, crystal packing, metal complexes, intermolecular charge transfer systems, cation–π complexation, electron conductivity, optical properties and so on. Keeping the importance of this beautiful scaffold in mind, we compiled all the synthetic routes available for the construction of sumanene and its heteroatom derivatives including Mehta’s first unsuccessful effort up to the latest achievements. Our major goal to write this review article was to provide a quick summary of where the field has been, where it stands at present, and where it might be going in near future. Although several reviews have been published on sumanene chemistry dealing with different aspects but this is the first report that comprehensively describes the ‘all-in-one’ chemistry of the sumanene architecture since its invention to till date. We feel that this attractive review article will definitely help the scientific community working not only in the area of organic synthesis but also in materials science and technology.

## Review

### 1 Introduction

Over a long period of time, polyaromatic hydrocarbons (PAHs) have attracted a tremendous attention of the scientific community because of their diverse potential applications ranging from the chemistry perspective to materials science and technology [1–4]. As we know that bowled (curved) surfaces are universal in nature for example our planets as well as atomic orbitals possess the curvature which generally affects the charge-transport, redox, self-assembly, and optical properties of bowl-shaped π-conjugated systems [5–9]. The synthesis of π-bowls is an extremely challenging job due to the presence of unusual strain in these types of molecules, therefore, the first synthetic breakthrough in this arena came into the picture in the late twentieth century when for the first time corannulene was reported by Barth et al. [10] at the University of Michigan (USA) in 1966 (crystal structure 1971) [11]. On the other hand, since the discovery of fullerene (C_60_) in 1985 [12] by Sir Harry Kroto and the first synthesis of sumanene in 2003 by Sakurai et al. [13], this field of research is continuously booming because of the developments of advanced synthetic organic tools [14–16]. Among the PAHs, the buckybowls are of significant importance not only because of the presence of unique inherent chirality (bowl-chirality) originating either from the bowl structure itself, e.g., in hemifullerene or by the introduction of substituents (e.g., trimethylsumanene) or heteroatoms (e.g., triazasumanene) into the achiral bowls but also as they are partial structures of carbon nanotubes (CNTs) and fullerenes having bowl inversion activities as well as a tendency of crystal packing [17–20]. Since these chiral buckybowls contain stable convex or concave faces suitable for the generation of chiral molecular recognition sites which can be used for the construction of helical assemblies and have been used to coordinate the metal atom(s) [21–23]. On the other hands, further extension of these molecules leads to homochiral carbon nanotubes which can produce innovative perception in chiral sensing, chiral catalysis, separation techniques and chiral ligands for organocatalysis [24]. Additionally, control on the bowl-to-bowl inversion can not only be useful for enantioselective synthesis of π-bowls but also to produce novel building blocks for molecular switches, chemical machines, molecular motor, ferroelectric memories, molecular devices, and sensory materials etc. Interestingly, electronic switching, thermal transport and thermoelectric properties in addition to the *onigiri*-type core-shell assemblies have been reported for sumanene and its derivatives. More interestingly, its application in the absorption of small molecules such as NH_3_, CO_2_, CO, and H_2_ using density functional theory (DFT) calculations has also been revealed [25–29].

**Figure 1:**
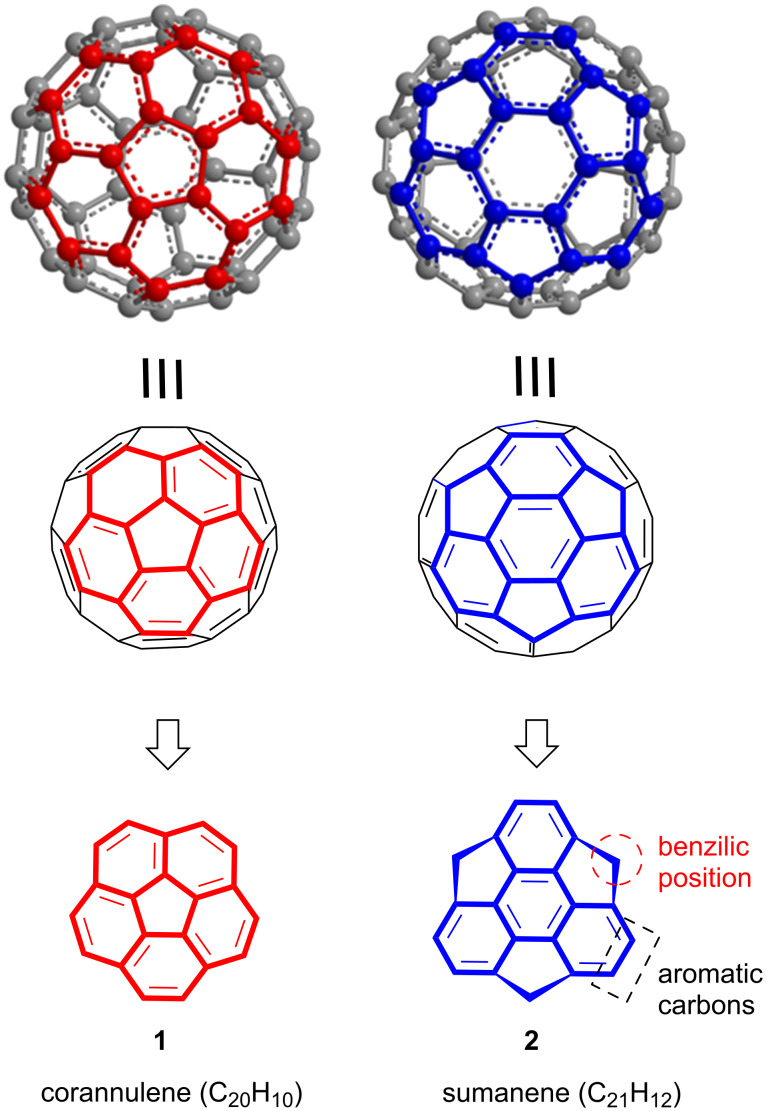
Representation of corannulene (**1**) and sumanene (**2**), the subunits of fullerene (C_60_).

Therefore, the area of buckybowls particularly sumanene chemistry is gaining more and more pace which can be seen by the inspection of a flow of publications appearing in the literature day-by-day [26]. As far as our knowledge is concerned, this is the first comprehensive review article which covers almost all the unsuccessful as well as successful efforts towards the synthesis of sumanene and its congeners. The ball-and-stick representation, bowl fragments and the chemical structures of corannulene (**1**) and sumanene (**2**) are displayed in the Figure 1.

### 2 Synthesis of sumanene and its derivatives

**Scheme 1:**
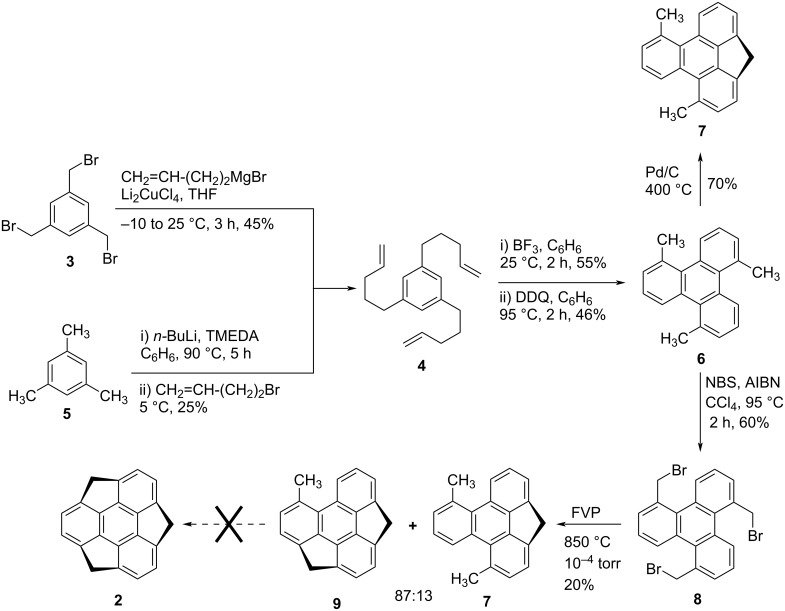
Mehta’s unsuccessful effort for the synthesis of sumanene scaffold **2**.

Around three decades before, it was the year 1993, when first time Mehta and his teammates coined the name sumanene for compound **2** from a Sanskrit word ‘Suman’ which means flower and put their unsuccessful effort towards the synthesis for this architecturally interesting molecule from 1,3,5-tris(bromomethyl)benzene (**3**) using the flash vacuum pyrolysis (FVP) technique as a key transformation (Scheme 1) [30]. As can be seen from an inspection of Scheme 1, they began their journey with the coupling reaction of **3** with 3-butenylmagnesium bromide in the presence of dilithium tetrachlorocuprate (Li_2_CuCl_4_) to produce the tripentenylbenzene derivative **4** in 45% yield. Alternatively, they have also prepared the same compound **4** starting from a simple and commercially available 1,3,5-trimethylbenzene (mesitylene) by using *n*-BuLi, and 4-bromo-1-butene in the presence of *N,N,N′,N′*-tetramethylethylenediamine (TMEDA) as shown in the Scheme 1. Having the compound **4** in hand, it was subjected to the cyclization in the presence of boron trifluoride to provide the tricyclohexyl-fused benzene derivative which on further dehydrogenation with 2,3-dichloro-5,6-dicyano-1,4-benzoquinone (DDQ) afforded 1,5,9-trimethyltriphenylene (**6**) in 46% yield. Later on, they first directly tried to convert compound **6** into the expected sumanene (**2**) by using a cyclodehydrogenation reaction in the presence of Pd/C at 400 °C, surprisingly they obtained only mono-bridged compound **7** in around 70% yield along with some of an unidentified mixture of compounds. In sharp contrast, their attempt under flash vacuum pyrolysis (FVP) at high temperature was also fruitless may be due to the presence of three benzylic sp^3^ carbons which are unable to endure such harsh reaction conditions. Therefore, next they converted compound **6** into 1,5,9-tribromomethyltriphenylene (**8**) using *N*-bromosuccinimide (NBS) in the presence of AIBN in CCl_4_. To their surprise, when compound **8** was subjected to FVP at around 850 °C temperature, only mono- and dibridged compounds (**7** and **9**) were isolated in overall 20% yield in (13:87) ratio. The structure of the dibridged compound **9** was not only confirmed by spectroscopic data but also identified by virtue of the single crystal structure. The reason for their unsuccessful results may be the generation of strain in the sumanene molecule from planar aromatic architecture under the experimental reaction conditions.

**Scheme 2:**
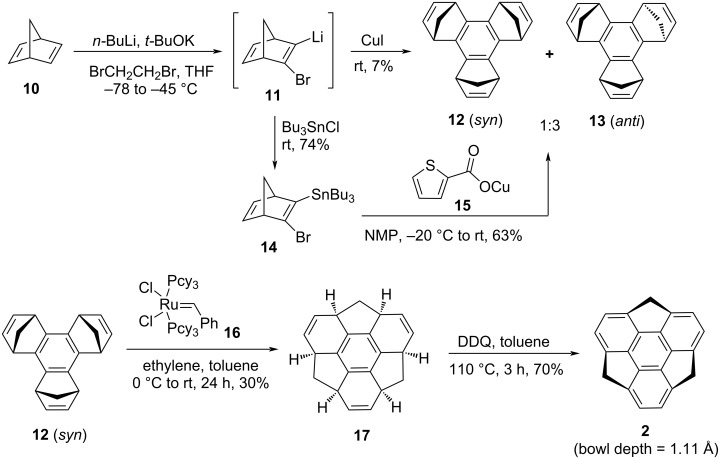
First synthesis of sumanene **2** by Sakurai et al. from norbornadiene **10**.

In the quest for developing the first successful synthetic method, groundbreaking news came from Sakurai’s laboratory around a decade after Mehta’s first unsuccessful attempt for the synthesis of sumanene (**2**, Scheme 2) [13]. Their exciting non-pyrolytic synthetic pathway for its construction commenced with an easily accessible compound, namely norbornadiene (**10**) by involving oxidative aromatization in the presence of DDQ via an intermediate **17**, as displayed in Scheme 2. As can be seen from an inspection of Scheme 2, they first performed a single step cyclotrimerization of **10** using *n*-BuLi and *t*-BuOK in 1,2-dibromoethane followed by the addition of CuI through an intermediate **11**. This procedure provided a very low yield (7%) of a mixture of **12** (*syn*) and **13** (*anti*) products. Therefore, they opted an alternative route which involves the formation of organotin compound **14** followed by trimerization in the presence of copper catalyst **15** to yield the trimerized products **12** (*syn*) and **13** (*anti*) in respectable yields (Scheme 2). The alkene-bridge exchange of **12** (*syn*) was accomplished by tandem ring-opening and ring-closing metathesis (ROM–RCM) in the presence of Grubbs’ first generation (G-I) catalyst to generate a *C*_3_-symmetric hexahydrosumanene **17** which on subsequent aromatization using DDQ furnished the desired molecule sumanene (**2**) in good yield. To their surprise, tandem metathesis for achieving compound **17** from **13** (*anti*) was fruitless may be because of the endothermic reaction by 37.4 kcal/mol as compared to the exothermic (51.4 kcal/mol) transformation of **12** (*syn*) to **17**, calculated by density functional theory (DFT) calculations.

**Scheme 3:**
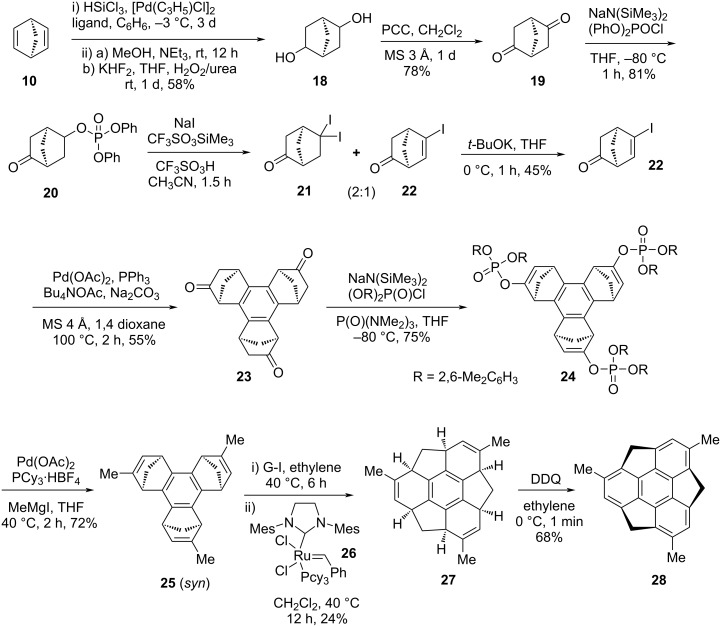
Synthesis of trimethylsumanene **28** from easily accessible norbornadiene (**10**).

On the other hand, in 2008, Higashibayashi et al. reported the synthesis of first chiral *C*_3_-symmetric trimethylsumanene **28** starting from enantiopure norbornadiene (**10**) by employing a rational synthetic strategy via the transfer of sp^3^ chirality of **27** into the bowl chirality of **28** as a key conversion (Scheme 3) [17,31]. In this context, they began with the Pd-catalyzed hydrosilylation reaction using HSiCl_3_ at −3 °C in the presence of a chiral phosphine ligand to furnish the hydrosilylated product which on subsequent Tamao–Fleming oxidation provided the *exo*-diol **18** in an overall good yield with 99% enantiomeric excess (Scheme 3). Furthermore, the diol **18** was converted into the corresponding diketone **19** using pyridinium chlorochromate (PCC) as an oxidizing agent. Interestingly, they have also performed the similar transformation in almost identical yield using Swern oxidation reaction conditions. The diketone **19** was then transformed into the enantiopure iodonorbornanone **22** in three steps which on further regioselective Pd-catalyzed cyclotrimerization furnished the *syn*-benzocyclotrimer **23** in 55% yield. Next, the methyl substituents were introduced at the position of the carbonyl groups of **23** to generate the methyl-substituted olefin derivative **25** via alkenyl phosphates **24** by means of three-fold cross-coupling reaction with methylmagnesium iodide in THF. Finally, during the tandem ROM–RCM, they noticed that the G-I catalyst provided a mixture of ring-opened products, therefore, the ring-closing step was then carried out using Grubbs’ second generation (G-II) catalyst. Hence, the tandem ROM–RCM step was made successful using both G-I and G-II catalysts in ethylene atmosphere to afford the *C*_3_-symmetric hexahydrotrimethylsumanene **27**, which was then converted into the desired compound by aromatization with DDQ. Alternatively, when they used the more active G-II catalyst instead of the G-I and then the G-II catalyst under similar reaction conditions, they directly observed the formation of compound **27** in a single step. They also pointed out that when ethylene gas was used as an alkene source for the ring-opening step, diluted reaction conditions were necessary to dissolve the ethylene in dichloromethane to avoid polymerization. Therefore, after several experimentations, they noticed that liquid *Z*-oct-4-ene in toluene was found to be a more effective alkene source for the ring-opening reaction in comparison to the gaseous ethylene, as it increases solubility and also improve the lifetime of the catalyst.

#### 2.1 Derivatization at benzylic position

**Scheme 4:**
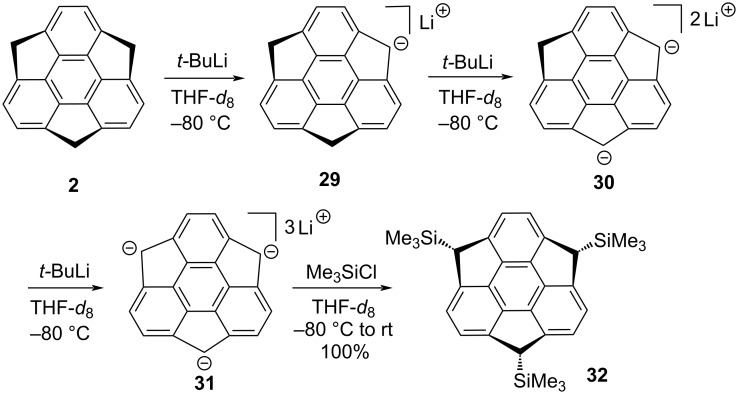
Generation of anions **29–31** and the preparation of tris(trimethylsilyl)sumanene **32**.

As can be inferred from the architecture of sumanene (**2**), it is not only gifted with three benzylic positions stapled between the benzene rings of the triphenylene system but also possess the bowl-curvature which enables stereoselective functionalization at these valuable positions. Taking the advantage of these benzylic positions, two years later to the trimethylsumanene synthesis, Sakurai and his co-workers selectively generated mono-, di- and trianions (**29–31**) using *t-*BuLi as a base and the formation of these sequential anions were confirmed by ^1^H and ^13^C NMR spectroscopy (Scheme 4) [32]. Next, the in situ generated trianion **31** was quenched with an excess of trimethylsilyl chloride (Me_3_SiCl) to furnish the only *exo-*tris(trimethylsilyl) derivative **32** may be because of the unhindered attack from the convex surface selectively in comparison to the hindered concave face (Scheme 4).

**Scheme 5:**
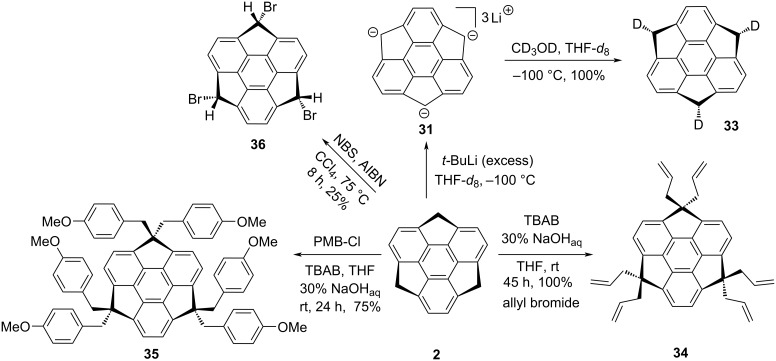
Synthesis of tri- and hexa-substituted sumanene derivatives.

Along similar lines, Hirao and his teammates have reported the trideuteriosumanene **33** in a stereoselective manner from the same trianion **31** just by trapping it with CD_3_OD/CH_3_OD (Scheme 5). In the same paper, they have also reported the hexaallylated sumanene **34** as well as hexa-*p*-methoxybenzylsumanene **35** by reacting sumanene (**2**) with allyl bromide and 4-methoxybenzyl chloride (PMB-Cl) in the presence of 30% NaOH_(aq)_ and tetrabutylammonium bromide (TBAB) in a minimum amount of THF (Scheme 5) [33]. On the other occasion, tribromosumanene **36** has also been synthesized via a radical mechanism from pristine sumanene (**2**) in the presence of NBS and CCl_4_ (Scheme 5) [14].

**Scheme 6:**
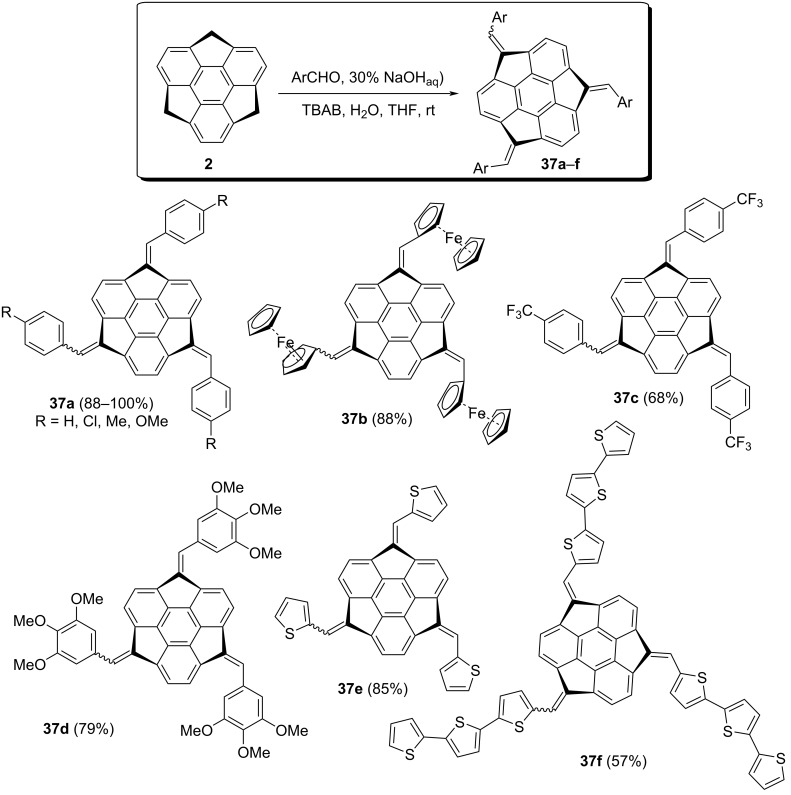
Synthesis of bowl-shaped π-extended sumanene derivatives **37a–f**.

In another study, the same group has also reported a series of both *C*_3_-symmetric as well as unsymmetric diastereomeric π-conjugated bowl-shaped molecules **37a–f** in good to quantitative yields (Scheme 6) [34–35]. These π-conjugated systems were assembled by treating sumanene (**2**) with different conjugated aromatic aldehydes using 30% aqueous NaOH in the presence of TBAB in THF. Although they have tried several bases for these condensation reactions the aforementioned base was found to be more effective than other bases used (Scheme 6). More interestingly, the sumanene derivative **37b** was used as a sensory material for selective recognition of cesium cations in water.

**Scheme 7:**
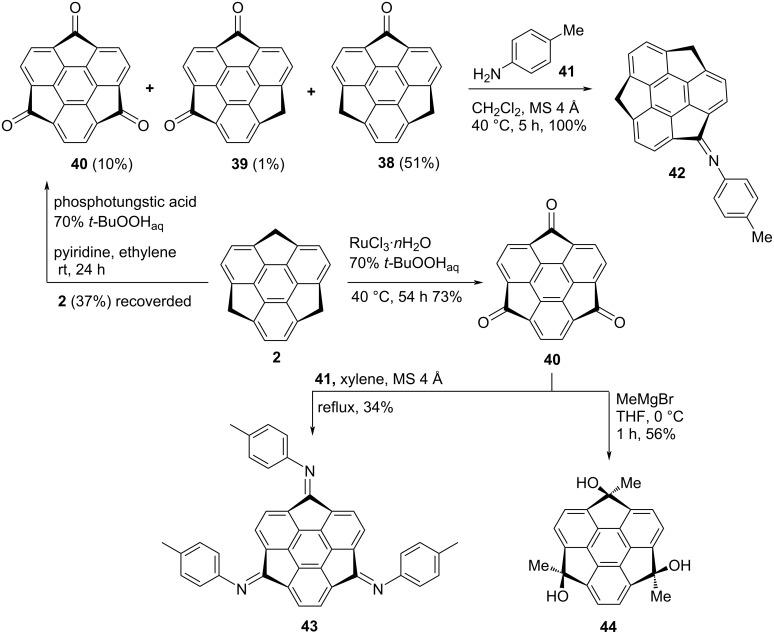
Synthesis of monooxasumanene **38**, trioxosumanene **40** along with imination of them.

In another event, Hirao’s group has prepared trioxosumanene **40** in the presence of RuCl_3_ and *t-*BuO_2_H which could be used as a key building block to generate diverse significant electroactive materials by virtue of nucleophilic addition reactions or by other means. After several experimentations, these workers were able to obtain trioxosumanene **40** in 73% yield (Scheme 7). More interestingly, monooxosumanene **38** was achieved selectively in good yield using phosphotungstic acid and *t*-BuO_2_H as shown in Scheme 7. To their surprise, they were unable to isolate dioxosumanene **39** under similar reaction conditions in reasonable yield. The structures of these compounds were established by spectroscopy means and also in the case of monosumanene **38**, they obtained the single crystal structure which was showing almost a similar bowl depth as for the parent sumanene (**2**). Later on, they transformed trione **40** into the corresponding *exo*-trimethyl derivative **44** by means of a Grignard reaction with MeMgBr via 1,2-addition (Scheme 7). The imination of monoketosumanene **38** as well as triketosumanene **40** has also been reported by these authors to obtain the corresponding compounds **42** and **43**, displayed in Scheme 7 [36–38].

**Scheme 8:**
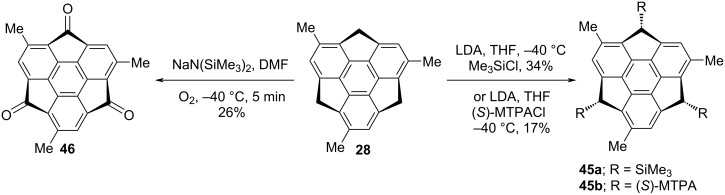
Synthesis of trimethylsumanenetrione **46** and *exo*-functionalized products **45a**,**b**.

Fascinatingly, the group of Sakurai has revealed the *exo*-functionalization of trimethylsumanene **28** to produce the products **45a**,**b** stereoselectively, as can be inspected from Scheme 8 [39]. The preparation of the compounds **45a**,**b** were achieved via the generation of trianions at the benzylic positions using LDA followed by the introduction of electrophilic partners such as (*S*)-MTPA (PhC(CF_3_)-(OMe)CO) and SiMe_3_ in THF to yield the required compounds in moderate yields (Scheme 8). The trimethylsumanenetrione **46** has also been generated from the corresponding trimethylsumanene **28** by means of nucleophilic oxidation using NaHMDS in the presence of molecular oxygen in DMF as the solvent (Scheme 8).

**Scheme 9:**
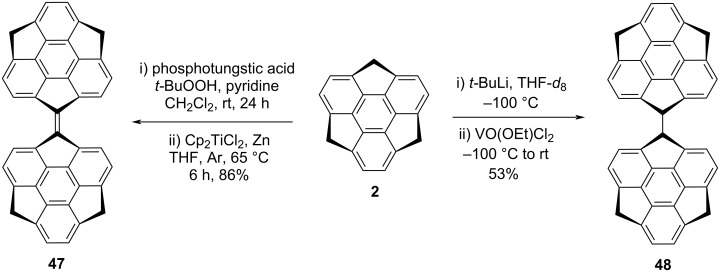
Synthesis of bisumanenylidene **47** and sumanene dimer **48** from **2**.

It has been noticed from the literature that the directly linked π-conjugated systems act as promising electron-accepting materials because of their high LUMO energy level and hence may be gifted to achieve a large open-circuit voltage. To this context, Amaya et al*.* has developed a novel bissumanenylidene **47** starting from the parent sumanene (**2**) first by converting it into the monoketosumanene followed by McMurry coupling reaction (Scheme 9). Although, they have attempted various combinations of Ti(IV) and reducing agents but Cp_2_TiCl_2_ with Zn powder provided the better results compared to the other combinations [14,40]. Additionally, they prepared benzyl–benzyl coupled sumanene dimer **48** by means of oxidative dimerization via sumanenyl monoanion intermediate **29** (Scheme 9) [14].

**Scheme 10:**
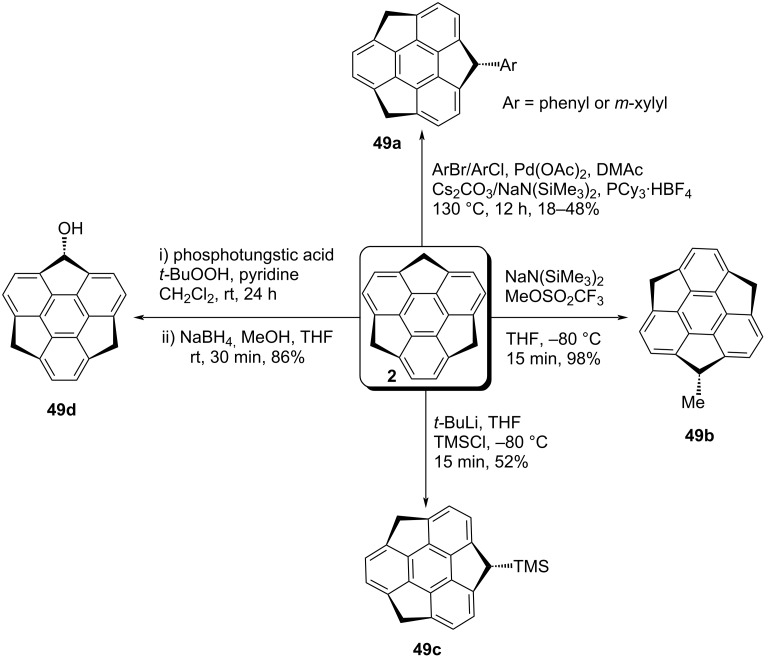
The mono-substitution of **2** to generate diverse mono-sumanene derivatives **49a–d**.

In 2012, Wu’s team have employed the Pd-catalyzed arylation reaction of sumanene with chlorobenzene and 2-bromo-1,3-dimethylbenzene to afford the aryl-substituted sumanenes **49a** (Scheme 10). On the other hand, Higashibayashi et al. has revealed the synthesis of three different mono-substituted sumanene derivatives **49b–d** in a stereoselective manner as can be inspected from Scheme 10 [41–42].

**Scheme 11:**
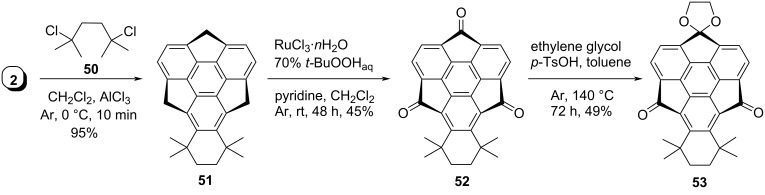
Synthesis of sumanene building block **53** useful for further extension.

In another event, Amaya, Ito, Katoh and Hirao reported a vital building block **53** to extend the π-conjugation bidirectionally through regioselective functionalization (Scheme 11) [43]. To achieve this goal, they commenced with the two-fold Friedel–Crafts alkylation reaction of sumanene (**2**) with 2,5-dichloro-2,5-dimethylhexane (**50**) involving AlCl_3_ to generate compound **51** which on subsequent oxidation provided triketosumanene **52** (Scheme 11). Finally, compound **52** was reacted with ethylene glycol to afford monocyclic acetal **53** with less hindered keto-group in respectable yield.

**Scheme 12:**
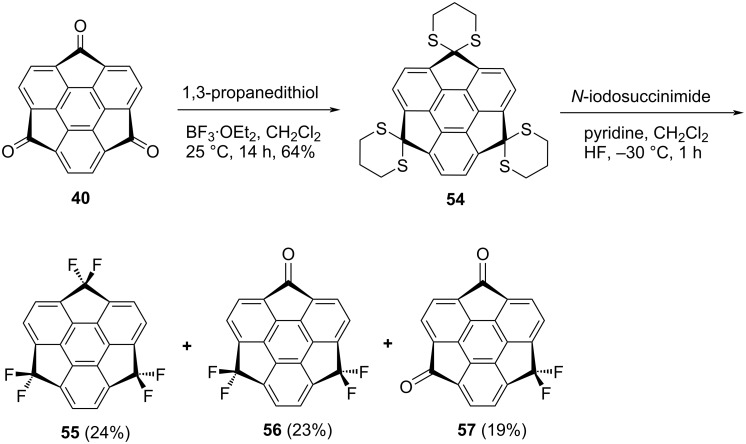
Synthesis of hexafluorosumanene derivative **55** by Sakurai and co-workers.

Since the introduction of powerful electron-withdrawing groups such as fluorine atom(s) in any material changes its behavior significantly, in this regard, Sakurai’s group installed six fluorine atoms at the benzylic positions of sumanene in a two-step approach to generate hexafluorosumanene **55** in an overall good yield [44]. As can be seen from an inspection of Scheme 12, they first prepared cyclic dithiane **54** from trisumanenone **40** using propanedithiol in the presence of BF_3_ since dithianes are outstanding precursors for the installation of fluorine atoms using appropriate fluorinating agents such as (difluoroiodo)benzene derivatives or elemental fluorine. In the second step, when they used *para*-iodotoluene difluoride the reaction conversion was very low, therefore, when using Olah’s reagent (pyridine hydrofluoride) in the presence of an activating agent, e.g., *N*-iodosuccinimide (NCS) in a usual glass flask, a mixture of products **55–57** were obtained (Scheme 12). Interestingly, when a polypropylene tube was used instead of glass flask, the expected hexafluorosumanene **55** was obtained in 73% yield.

**Scheme 13:**
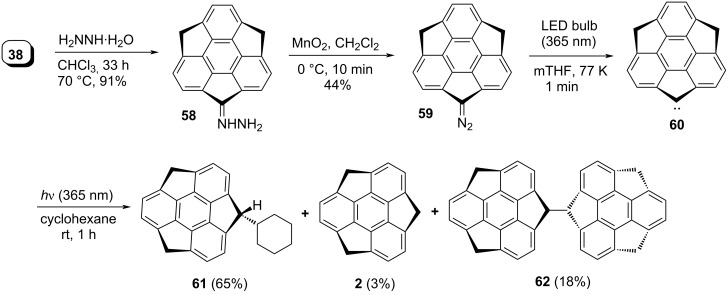
Preparation of sumanene-based carbene **60** and its reaction with cyclohexane.

**Scheme 14:**
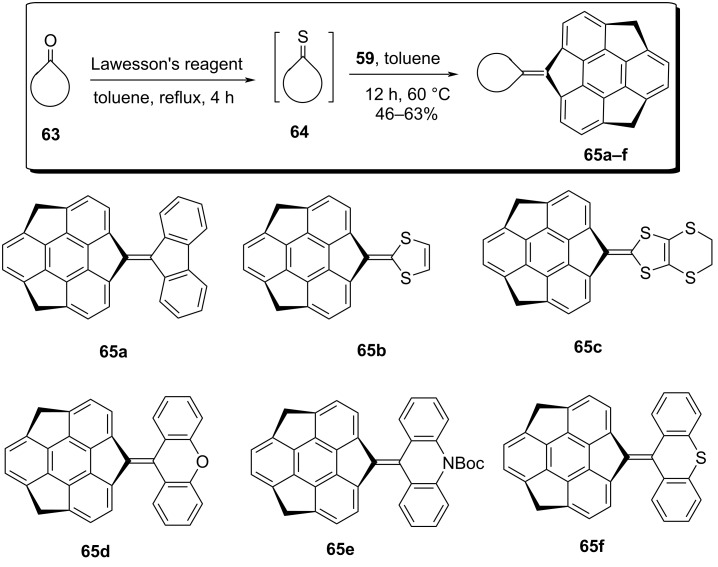
Barton–Kellogg reaction for the synthesis of sterically hindered alkenes.

To explore the chemistry of the sumanene and its congeners, quite recently the same group has also prepared sumanene-based carbene **60** starting from monosumanenone **38** by reacting it with hydrazine hydrate to provide the corresponding hydrazone **58** which on further oxidation with MnO_2_ followed by irradiation using an LED lamp at ca. 365 nm for 1 min in 2-methyltetrahydrofuran (mTHF) at 77 K gave **60** (Scheme 13) [45]. It was noticed from both experimental as well as theoretical studies that the ground state of the prepared carbene **60** is a triplet which was confirmed by ESR as well as density functional theory (DFT) calculations. As we are aware that if the carbene formed is a singlet then a C–H bond inserted product is predominating whereas if the dimer is the major product along with the minor C–H bond inserted product then the triplet carbene is generated. During their study, they obtained the C–H inserted product **61** in 65% along with the dimer **62** in 18% yield with cyclohexane, confirming that the reaction precedes via the singlet state, albeit it was a triplet species in the ground-state. They further reported the applicability of the carbene precursor **59** by reacting it with different thiocarbonyl compounds in a Barton–Kellogg coupling manner to yield the corresponding olefins. To this context, the carbonyl species were first transformed into the thiocarbonyl systems in the presence of Lawesson’s reagent which were then reacted with diazosumanene **59** to furnish the corresponding alkene systems. It has been noticed from the literature that the construction of these types of sterically hindered tetrasubstituted alkenes is really difficult through McMurry coupling reaction or by other means (Scheme 14).

#### 2.2 Functionalization at benzene ring(s) bay positions

**Scheme 15:**
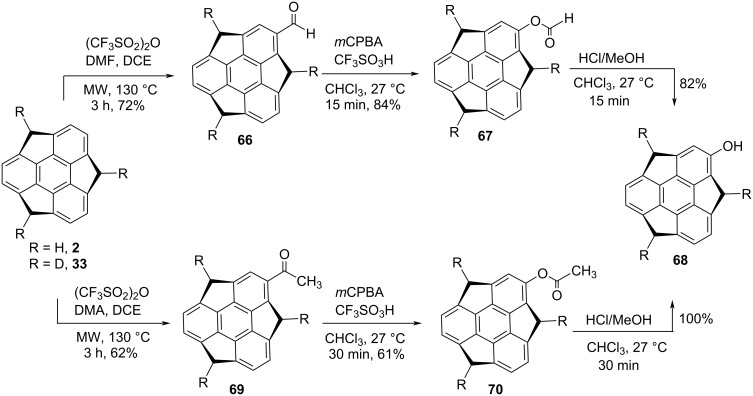
Synthesis of hydroxysumanene **68** by employing Baeyer–Villiger oxidation.

In another event, the Sakuria group has also reported hydroxysumanene **68** by means of a Baeyer–Villiger oxidation reaction of acyl- or formylsumanene derivatives in the presence of *m*-chloroperbenzoic acid (*m*-CPBA) followed by acid-catalyzed solvolysis in 10% HCl/MeOH (Scheme 15) [46]. Interestingly, it was found from both theoretically as well as experimentally that compound **68** has a deeper bowl architecture and also a higher bowl inversion energy as compared to the sumanene (**2**), measured by NMR studies. In this report, these workers further revealed the electronic state of **68** by means of electrochemical and UV-absorption measurements.

**Scheme 16:**
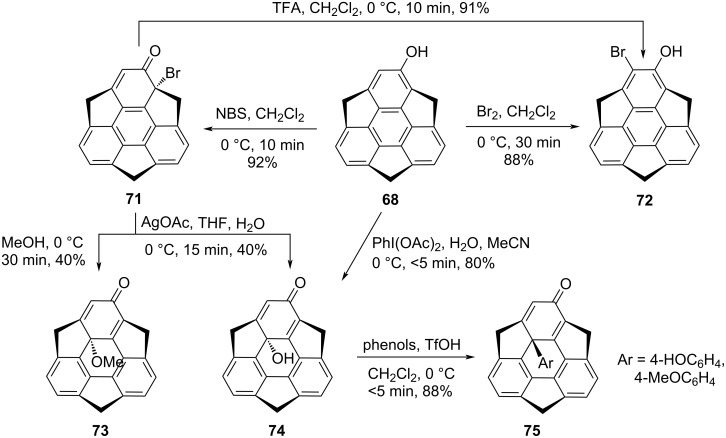
Synthesis of sumanene derivatives having functionality at an internal carbon.

**Scheme 17:**
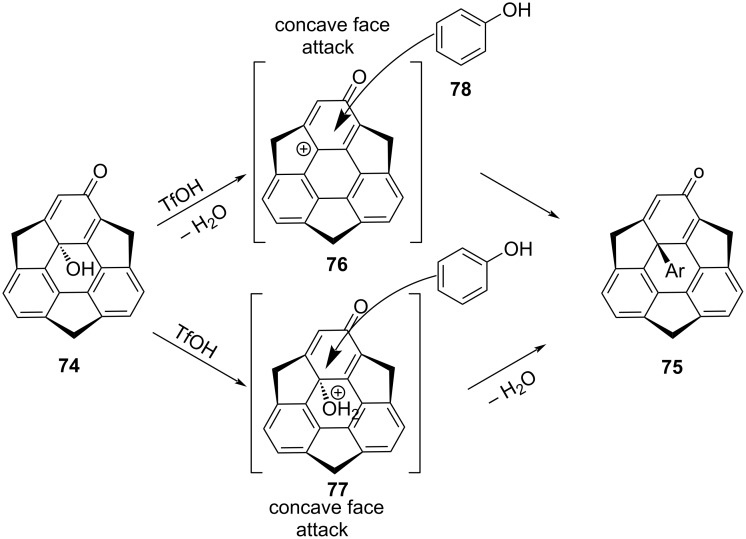
Mechanism for nucleophilic substitution reaction at the internal carbon.

Recently, they have also reported the functionalized sumanene derivatives **73**–**75** at the convex side of the internal carbon commencing from the bromination of the hydroxysumanene **68** using NBS and subsequent nucleophilic substitution reaction [47–48]. To their surprise, when they used molecular bromine instead of NBS, they obtained *o*-bromohydroxysumanene **72** instead of **71**. More interestingly, it was also noticed that by treating **71** with trifluoroacetic acid (TFA), it instantly transformed into sumanene derivative **72**, suggesting that **71** is unstable under acidic conditions. The acid-catalyzed aromatization can be explained via the migration of the bromide ion followed by the aromatization of bromodienone compound **71** to the stable *o*-bromohydroxysumanene **72**. Having compound **71** in hand, next it was treated with silver acetate in H_2_O/THF (1:1) to furnish compound **74**. On the other hand, alcoholysis of the same compound **71** with methanol provided methoxysumanenone **73** in an overall good yield (Scheme 16). From their studies they observed that compound **74** is unstable to both heat as well as light, may be due to instability of the internal C–O bond. In this regard, very recently they transformed this unstable compound **74** into the stable derivatives **75** having a strong C–C bond through the nucleophilic substitution reaction with phenol and anisole in the presence of trifluoromethanesulfonic acid (TfOH) with total stereoinversion. This suggests that the nucleophile attacks occur from the concave face of the π-bowl [48]. Although, for the nucleophilic substitution reaction with phenol derivatives, they have tried several reaction conditions including the amount of acid as well as phenols. After several experimentations, they found that 30 equivalents of phenol and 1 equivalent of TfOH at 0 °C provided the best results. As can be inspected from Scheme 16, in this report, they have also developed an alternate route for the synthesis of compound **74** involving only one step instead of the previously reported two step approach. The possible mechanism for the nucleophilic substitution at the internal carbon is displayed in Scheme 17.

**Scheme 18:**
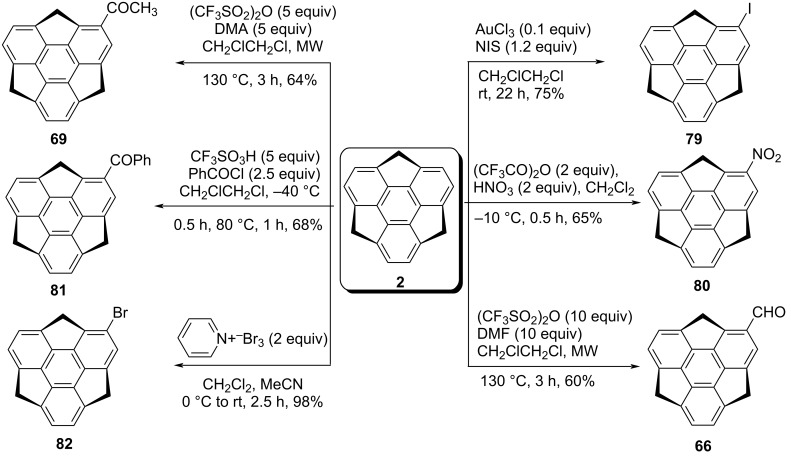
Synthesis of diverse monosubstituted sumanene derivatives.

**Scheme 19:**
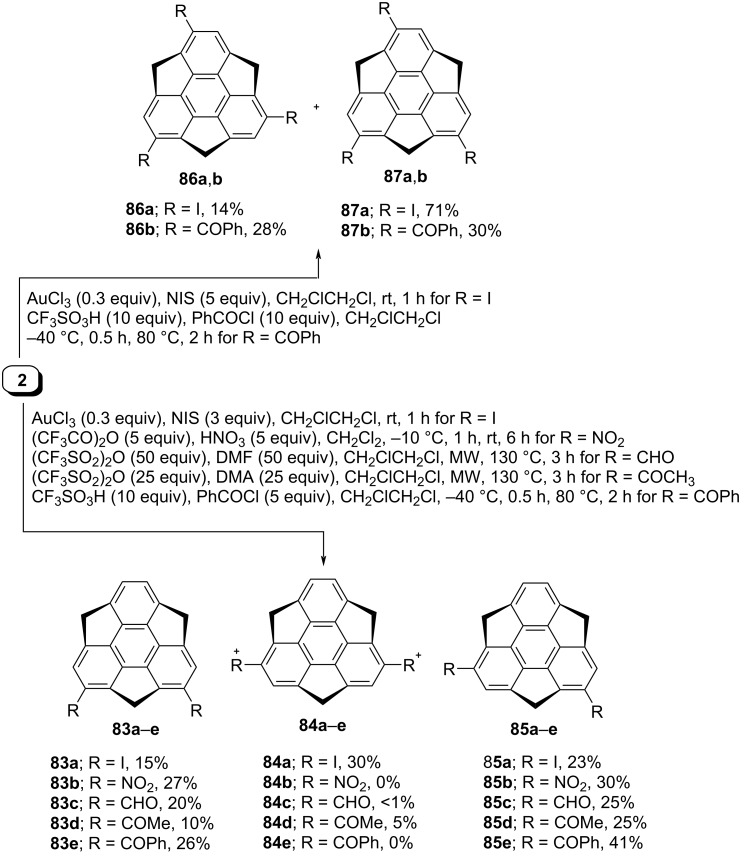
Synthesis of di- and trisubstituted sumanene derivatives from sumanene (**2**).

Amaya et al. in 2009 have revealed the synthesis of monobromosumanene **82** from sumanene (**2**) by treating it with pyridinium perbromide as displayed in Scheme 18 [49]. In this report, the authors have exposed the anisotropic electron transport properties of the needle-like single crystal of sumanene derivative **82** by means of time-resolved microwave conductivity technique. On the other hand, four years later, Sakurai and his teammates have reported the selective synthesis of diverse monosubstituted sumanene derivatives by employing electrophilic aromatic substitution reactions as they are the most trustworthy strategies for direct functionalization of aromatic scaffolds [50]. As can be inspected from Scheme 18, monoiodosumanene **79** was obtained by gold-catalyzed iodination in the presence of *N*-iodosuccinimide (NIS). The mononitrosumanene **80** was achieved through the nitration using trifluoroacetyl nitrate which was in situ generated from conc. HNO_3_ and trifluoroacetic anhydride. On the other hand, formylation and acetylation were performed under microwave conditions at 130 °C using triflic anhydride and DMF or dimethylacetamide (DMA) to deliver the corresponding monoformylsumanene **66** and monoacetylsumanene **69** in good yields. The monobenzoylsumanene **81** was prepared from benzoyl chloride in the presence of triflic acid. Moreover, disubstituted sumanene derivatives **83–85** were prepared under similar reaction conditions just by increasing the amount of the reagents and easily separable regioisomers were obtained in moderate-to-good overall yields (Scheme 19). In similar fashion, trisubstituted sumanene derivatives were also prepared as shown in Scheme 19.

**Scheme 20:**
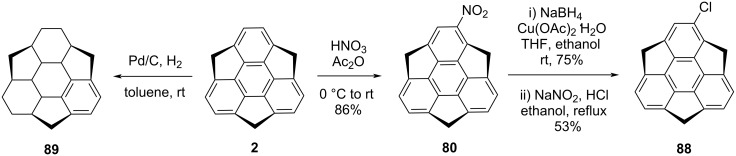
Preparation of monochlorosumanene **88** and hydrogenation of sumanene (**2**).

The monochlorosumanene **88** was also reported by Amaya and Hirao in three steps from the sumanene (**2**) using a classic nitration reaction followed by the reduction of the nitro functionality into the amine group and subsequently performing a Sandmeyer reaction as shown in Scheme 20 [14]. Additionally, they have reported the hydrogenation of sumanene to generate compound **89** having one benzene ring intact in the framework using Pd/C in the presence of hydrogen gas in toluene at room temperature, confirmed by mass spectral data as well as NMR spectroscopy (Scheme 20) [14].

**Scheme 21:**
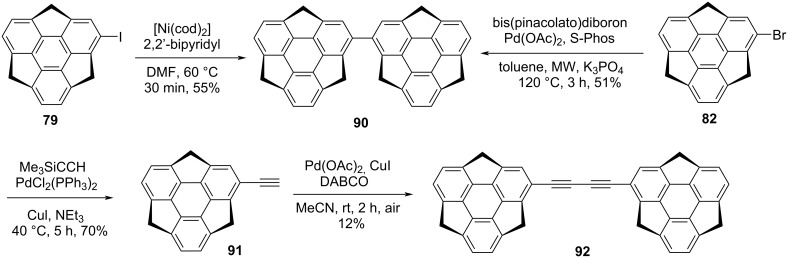
The dimer **90** and bissumanenyl **92** achieved from halosumannes.

As can be inspected from Scheme 21, the dimer **90** of sumanene (**2**) was obtained by two different routes, first by employing a one-pot borylation as well as a Suzuki–Miyaura cross-coupling reaction of bromosumanene **82** under microwave reaction conditions. Whereas another route involves the Ni-catalyzed aryl–aryl homo-coupling between the two molecules of iodosumanene **79** [51–52]. Moreover, the bissumanenyl **92**, which is thought to be chiral because of the two connected asymmetric bowls, as well as atropisomerism, was constructed by Hirao and his two group members, namely Amaya and Kobayashi, starting from the same bromo derivative **82**. First it was converted into ethynylsumanene **91** using a Sonogashira-coupling which on subsequent desilylation and Glaser-coupling reaction yielded **92** (Scheme 21).

**Scheme 22:**
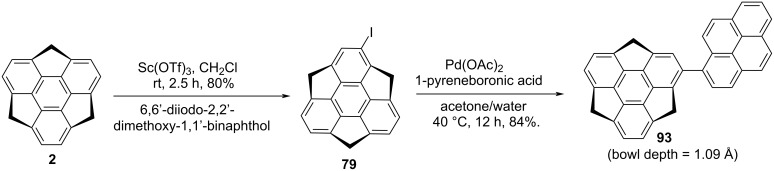
Pyrenylsumanene **93** involving the Suzuki-coupling as a key transformation.

As can be seen from the literature, pyrene-based compounds remain among the highest beautiful and fascinating classes of molecules because of their extremely characteristic (“fingerprint”) optical absorption as well as emission behavior. Keeping in mind the uniqueness of the pyrene moiety, an appealing sumanene derivative **93**, namely as pyrenylsumanene, was constructed by the groups of Higashibayashi and Sakurai which display both herringbone and columnar crystal packing (Scheme 22) [53]. To synthesize this architecturally interesting molecule, they began with the monoiodosumanene **79**, which was prepared via an alternate route using 6,6’-diiodo-2,2’-dimethoxy-1,1’-binaphthol in the presence of Sc(OTf)_3_. The Suzuki-coupling reaction between 1-pyreneboronic acid and iodosumanene **79** furnished the desired compound **93** in 84% yield (Scheme 22).

**Scheme 23:**
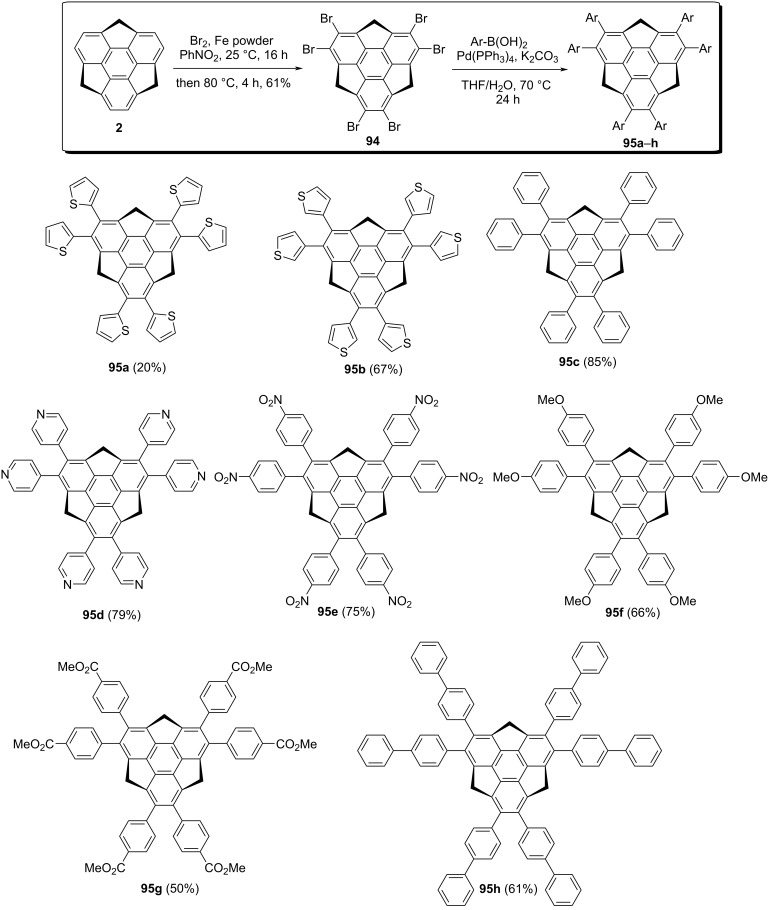
Synthesis of various hexaarylsumanene derivatives using the Suzuki-coupling reaction.

Among the bowl-shaped π-conjugated molecules, sumanene is more attractive as chemical modifications can be easily performed at both benzylic as well as peripheral aromatic carbons. Although, a handful of reports are available in the literature related to the selective functionalization at the benzylic carbons of the sumanene. In contrast, very few reports could be found for selective functionalization at the peripheral carbons because of difficulties arose during their synthesis. In this regard, Toda et al*.* in 2017 have successfully synthesized 2,3,5,6,8,9-hexaarylsumanene derivatives **95a–h** utilizing a Suzuki–Miyaura cross-coupling reaction as the critical step [54]. Towards this goal, they started with the hexabromination of sumanene using bromine and iron powder in nitrobenzene to provide the desired compound **94** in 61% yield. Having the bromosumanene **94** in hand, it was then subjected to the Suzuki coupling with several arylboronic acids in the presence of Pd(PPh_3_)_4_ and K_2_CO_3_ in THF/water to furnish the required hexaarylated sumanenes in 20–85% yields (Scheme 23).

**Scheme 24:**
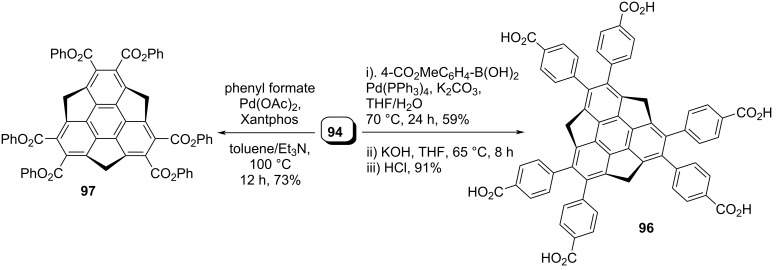
Synthesis of hexasubstituted sumanene derivatives **96** and **97**.

In the same year, the groups of Hisaki, Sato and Sakurai have reported a hydrogen-bonded 2D sumanene buckybowl framework having 4,4’-dicarboxy-*o*-terphenyl groups in the periphery. Thy employed the Suzuki coupling as the crucial step (Scheme 24) [55]. In contrast, very recently, Sakurai’s group has described the synthesis of 2,3,5,6,8,9-hexakis(phenoxycarbonyl)sumanene (**97**) from hexabromosumanene **94** as depicted in Scheme 24 [56]. Because of the low solubility of **94** in common organic solvents, they first tried the esterification reaction with soluble monoiodosumanene **79** using Pd(OAc)_2_ under CO atmosphere and obtained the desired sumanene methyl ester in very low yield. Surprisingly, when they used hexabromosumanene **94** under similar reaction conditions, no product formation was observed. Therefore, they used a Pd-catalyzed carbonylative esterification in the presence of phenyl formate which in situ generated CO and phenol to provide the required product **97** in 73% yield (Scheme 24).

**Scheme 25:**
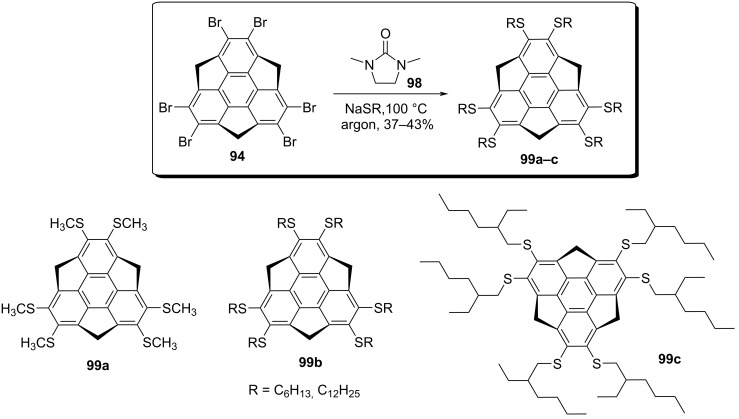
Synthesis of thioalkylsumanenes *via* an aromatic nucleophilic substitution reaction.

In an independent work reported in 2017, Fukushima and co-workers detailed the synthesis of first liquid-crystalline sumanene derivatives **99a–d** accomplished by the incorporation of six thioalkyl groups in the peripheral aromatic positions through aromatic nucleophilic substitution reaction of hexabromosumanene **94** with thioalkoxide (Scheme 25) [57]. As can be inspected from Scheme 25, an excess of sodium thioalkoxide in 1,3-dimethyl-2-imidazolidinone **98** reacted with **94** at 100 °C to generate the required sumanene derivatives **99a–c**.

In another report, Sakurai’s group in situ generated sumanyne and treated it with different dienes (**101a–d)** in a Diels–Alder (DA) manner to produce the corresponding DA adducts **102a–d** in moderate yields as shown in Table 1 [58]. *o*-Bromohydroxysumanene **72** was converted to *o*-hydroxysumanenyl borate **100** by means of a Pd-catalyzed Miyaura-borylation reaction. Compound **100** was then subjected to triflation and subsequent treatment with CsF afforded sumanyne which on further reaction with dienes furnished the DA-adducts **102a–d**.

**Table 1:** Synthesis of sumanene-based DA adducts through sumanyne intermediate.

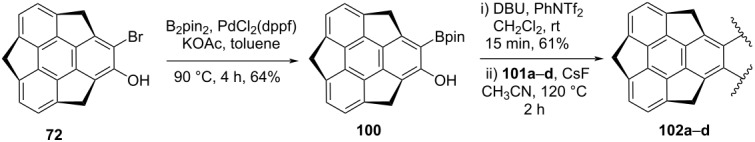			

Entry	Diene	Product	Yield [%]

1	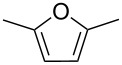 **101a**	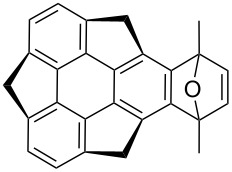 **102a**	53
2	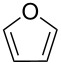 **101b**	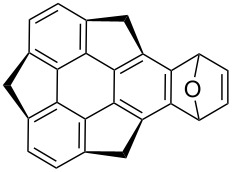 **102b**	40
3	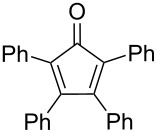 **101c**	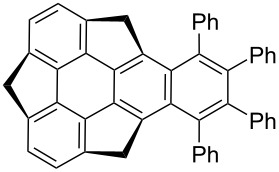 **102c**	36
4	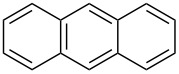 **101d**	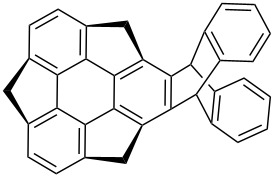 **102d**	22

**Scheme 26:**
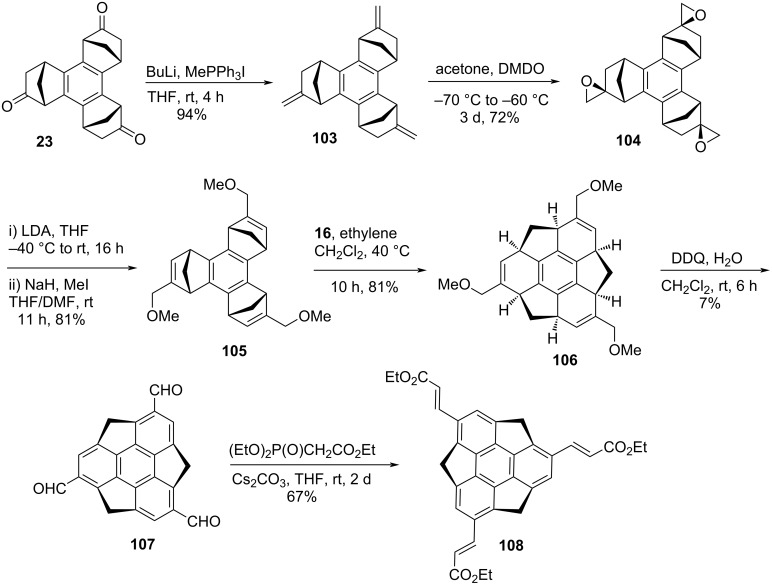
Synthesis of tris(ethoxycarbonylethenyl)sumanene derivative **108**.

In 2012, another type of functionalized sumanene, namely tris(ethoxycarbonylethenyl)sumanene **108** was prepared by Sakurai's group through ROM–RCM as well as Horner–Wadsworth–Emmons (HWE) reactions as key transformations (Scheme 26) [59]. To this context, their journey started from *syn*-tris(oxonorborneno)benzene **23**, obtained via palladium-catalyzed cyclotrimerization of iodonorbornene. The *C*_3_-symmetric compound **23** was then converted into the methylene groups containing compound **103** using Wittig reaction which on further treatment with dimethyldioxirane (DMDO) gave the triepoxy compound **104** in decent yield. Next, the methoxymethyl groups were installed by means of ring-opening reaction of **104** with LDA followed by protection of thus-created hydroxy groups in the presence of NaH/MeI to afford compound **105**. Compound **105** was then transformed into **106** by virtue of ROM–RCM which on subsequent DDQ oxidation followed by HWE reaction provided the desired compound **108** (Scheme 26).

**Scheme 27:**
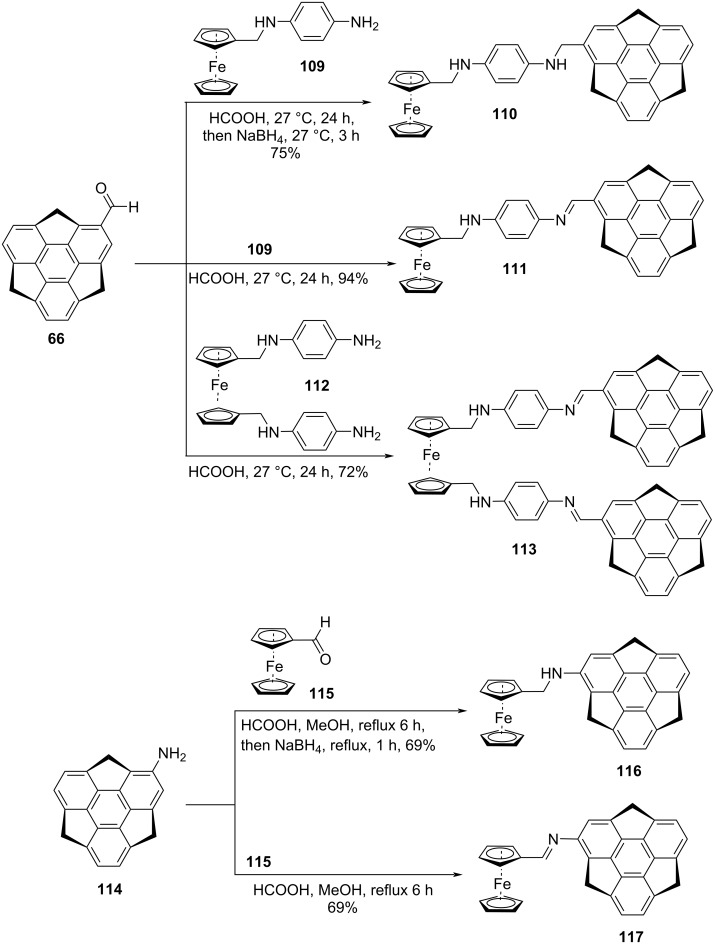
Synthesis of ferrocenyl-based sumanene derivatives.

In a really brilliant manner, Kasprzak and Sakurai in 2019 have created a sumanene–ferrocene dual system for selective recognition of cesium cations by means of site-selective cation–π interaction (Scheme 27) [60]. In this study, they used already discussed formylsumanene **66** and aminosumanene **114** prepared from nitrosumanene through the reduction of **80** with Pd/C/H_2_. As can be seen from Scheme 27, these attractive building blocks **66** and **114** were then converted into the ferrocene–sumanene conjugates by employing reductive amination reaction for the formation of compounds **110** and **116**. Whereas compounds **111**, **113**, and **117** were prepared by means of condensation reaction as depicted in Scheme 27.

**Scheme 28:**
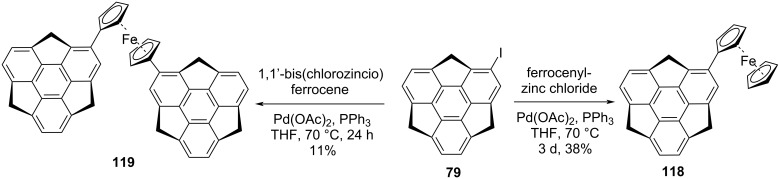
Synthesis of sumanenylferrocene architectures **118** and **119** *via* Negishi coupling.

On the other hand, Lentz’s group has reported the synthesis of sumanenylferrocene **118** as well as 1,1’-disumanenylferrocene **119** through Pd-catalyzed Negishi coupling of iodosumanene (**79**) with ferrocenylzinc chloride and 1,1’-bis(chlorozincio)ferrocene (Scheme 28) [9]. These synthesized molecules were confirmed utilizing spectroscopic techniques as well as by virtue of X-ray crystallographic analysis.

**Scheme 29:**
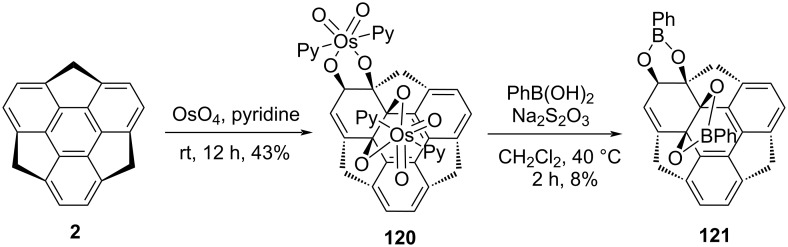
Diosmylation and the synthesis of phenylboronate ester **121** of sumanene.

To further advance the sumanene chemistry, in 2018, Sakurai and co-workers reported diosmylation and also synthesized the phenylboronate ester **121** of sumanene (**2**, Scheme 29) [24]. The osmylation of sumanene was carried out by OsO_4_ in pyridine to yield the diadduct **120**. Although, they tried to obtain the monoadduct by decreasing the amount of OsO_4_ but all the efforts produced only diadducts along with unreacted sumanene (**2**). Later on, to confirm the configuration, they attempted to transform it into the tetrol through the decomplexation of the osmate ester using Na_2_S_2_O_3_ and *t*-BuOH/H_2_O solution, but they received unsuccessful results may be due to its unstable behavior. Pleasingly, they got the fruitful result by converting **120** into phenylboronate ester **121** as can be noted from an inspection of Scheme 29.

#### 2.3 Complexation of sumanene and its derivatives

**Scheme 30:**
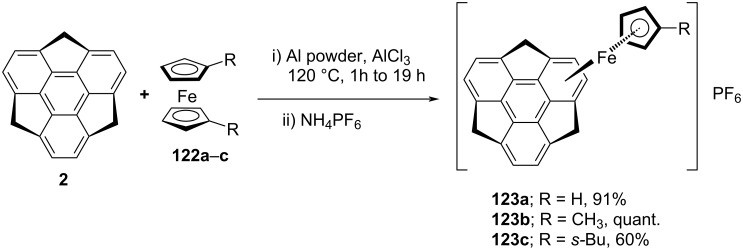
Synthesis of the iron-complex of sumanene.

Since coordination is one of the vital modes in metal binding and a range of π-conjugated planar systems having η*^n^*-binding to metals have been reported. In contrast, buckybowls have multiple coordination sites for instance the positions available in the polycyclic architecture and also because of the presence of concave or convex faces. Therefore, in recent years, the coordination of bowl-shaped molecules with the transition metals is of fundamental interest in the area of π-bowls chemistry since the first details of the metal complex of C_60_. In this context, Hirao’s group has reported the first example of Fe(η*^6^*-sumanene) complex **123** as an *endo*-coordinated complex at the concave side. The complex **123a** was prepared through the metalation of sumanene involving ligand exchange with a cyclopentadienyl (Cp) group of ferrocene by heating in the presence of Al powder and AlCl_3_ under solvent-free conditions followed by a counter-anion exchange with hexafluorophosphate (Scheme 30) [61]. Two years later to this report, the same group has also reported the synthesis of complexes **123b** and **123c** under almost similar reaction conditions [62].

**Scheme 31:**
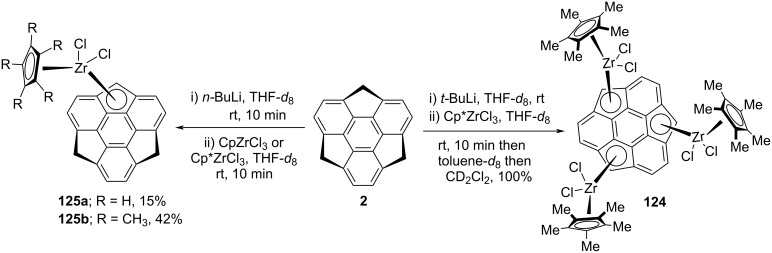
Synthesis of tri- and mononuclear sumanenyl zirconocene complexes.

**Scheme 32:**
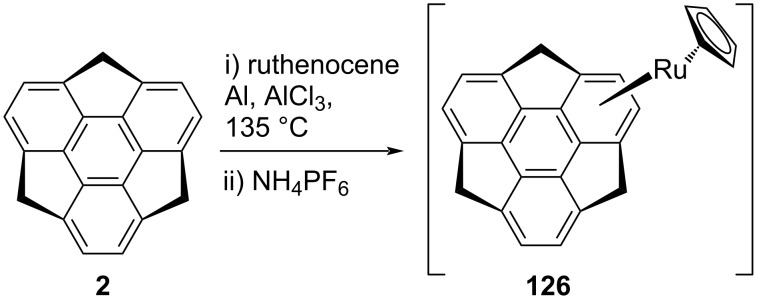
Synthesis of [CpRu(η^6^-sumanene)]PF_6_.

On the other occasion, they have also prepared the tri- and mononuclear sumanenyl zirconocene complexes **124** and **125**, respectively, as displayed in Scheme 31 [63]. The mono-zirconocene complex **125** was arranged by treating sumanene (**2**) with 1 equivalent of *n*-BuLi in THF-*d*_8_ to generate the monoanion at the benzylic position which on further treatment with CpZrCl_3_ provided the mono-zirconocene complex **125a** as a brown solid. Alternatively, when the monoanion was reacted with Cp*ZrCl_3_ under similar reaction conditions, the complex **125b** was achieved as a red solid (Scheme 32). The trinuclear sumanenyl zirconocene **124** was prepared by treating sumanene (**2**) with 3.1 equivalents *t*-BuLi in THF-*d*_8_ followed by quenching with Cp*ZrCl_3_ to furnish the blackish-green solution. Since the complex **124** was found to be unstable in THF-*d*_8_, therefore, immediately it was replaced by toluene-*d*_8_ which was further replaced by CD_2_Cl_2_ to remove THF-*d*_8_ completely.

Furthermore, they reported the ruthenium sumanene complex [CpRu(η^6^-sumanene)]PF_6_ **126** along with its bowl-to-bowl inversion and anticipated it to be more flexible in comparison to the iron analogue **123a**, may be due to the longer C–Ru bond (Scheme 32) [64]. The complex **126** was prepared in a similar manner as its iron analogue was prepared.

**Scheme 33:**
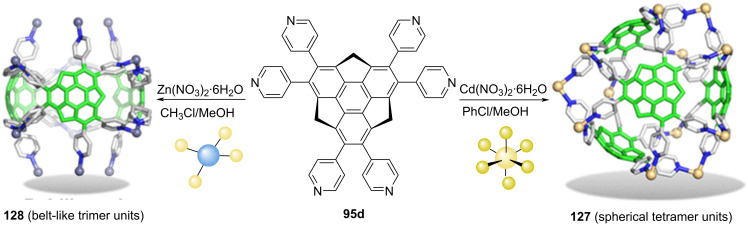
Preparation of sumanene-based porous coordination networks **127** (spherical tetramer units) and **128** (belt-like trimer units) (reproduced with permission from [65]. Copyright 2019 American Chemical Society).

Quite recently, Yakiyama, Hasegawa, and Sakurai reported two sumanene-based porous coordination networks **127** (spherical tetramer units) and **128** (belt-like trimer units**)** through complexation of the hexapyridylsumanene **95d** with Cd(NO_3_)_2_·4H_2_O as well as Zn(NO_3_)_2_·6H_2_O, (Scheme 33) [65]. The *C*_3_-symmetric sumanene derivative **95d** was assembled through Suzuki reaction of hexabromosumanene with 4-pyridylboronic acid as detailed in the above section. Having the hexapyridylsumanene **95d** in hand, it was dissolved in MeOH/CHCl_3_ solution and then the MeOH solution of Zn(NO_3_)_2_·6H_2_O was diffused into it for several days to afford colorless crystals of **128**. Along similar lines the crystals of **127** were achieved by following the same procedure.

**Scheme 34:**
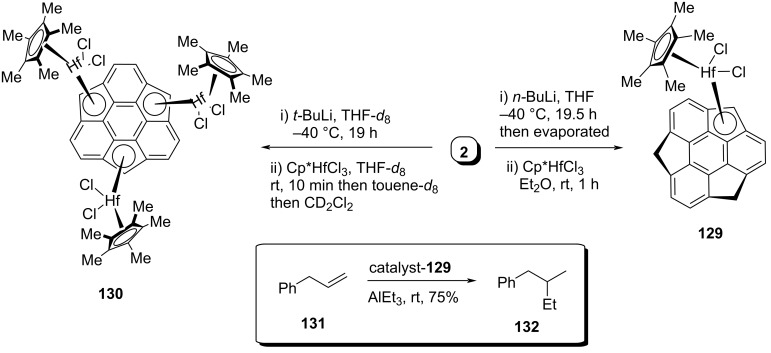
Synthesis of sumanenylhafnocene complexes **129** and **130**.

On the other hand, the groups of Amaya and Hirao have reported the synthesis of sumanenyl mono- and trihafnocene complexes **129** and **130**, respectively, along with the catalytic activity of **129** for hydroethylation of allylbenzene through carboalumination (Scheme 34) [66]. Complex **129** was prepared by treating pristine sumanene (**2**) with *n*-BuLi to generate the monoanion which on further treatment with Cp*HfCl_3_ provided the required monohafnocene complex **129**. Alternatively, to obtain the trianion species, they subjected **2** with an excess of *t*-BuLi and then quenched it with Cp*HfCl_3_ as displayed in Scheme 34. Furthermore, they revealed for the first time the catalytic activity of sumanene metallocene for the hydroethylation reaction of unactivated allylbenzene by treating it with AlEt_3_ at room temperature to furnish compound **132** (Scheme 34).

**Scheme 35:**
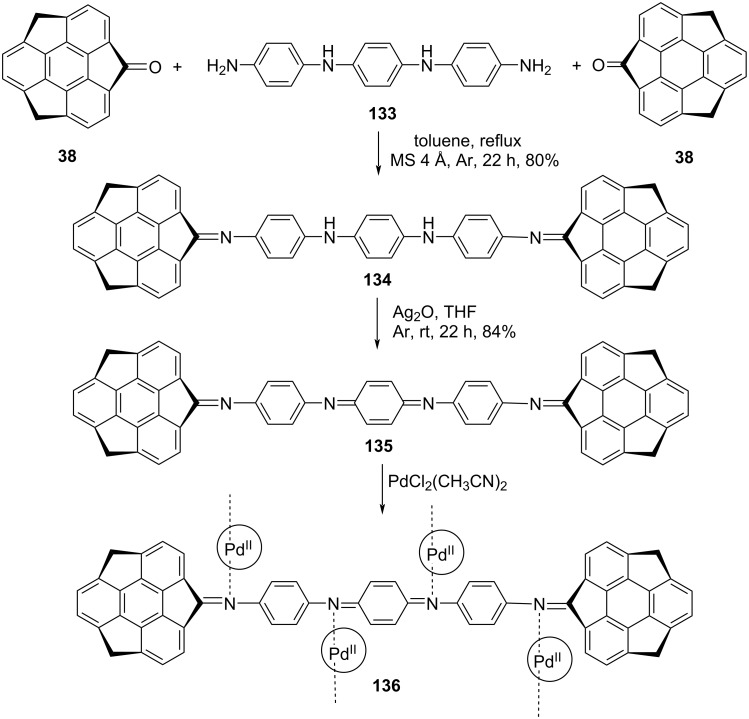
Synthesis of **134** and **135** along with Pd^II^ coordination complex **136**.

Since polyanilines are interesting π-conjugated polymers that contain characteristic conductivity by acid doping, environmental stability, optical and redox properties. Therefore, bridging of oligoaniline as a spacer between the functional units can lead toward the generation of versatile functional organic materials. On the basis of this concept, in recent years, a handful of appealing molecules and materials have been synthesized and due to which nanotechnology as well as supramolecular chemistry are continuously attracting the recent attention of researchers worldwide. To this context, Hirao’s team has reported the synthesis of sumanenemonoone imine compounds **134** and **135** along with the Pd(II) complex **136** formed in a stepwise coordination manner investigated by a titration experiment (Scheme 35) [67]. As can be inspected from Scheme 35, compound **134** was prepared by condensing sumanenone **38** with the amino-system **133** in refluxing toluene. The synthesized compound **134** was then treated with Ag_2_O in THF to afford the corresponding quinonediimine **135** in respectable yield. Furthermore, the stepwise coordination of the imino functionality of compound **135** to the palladium(II) was carried out in the presence of PdCl_2_(MeCN)_2_ in a stepwise manner, confirmed by UV–vis spectroscopic technique.

**Scheme 36:**
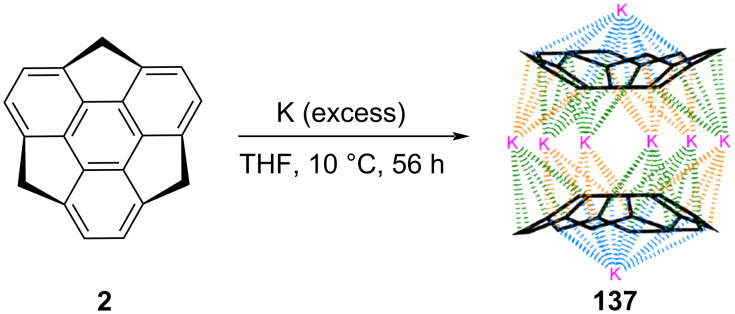
Synthesis of alkali metals sumanene complex K_7_(C_21_H_102_^−^)_2_(C_21_H_93_^−^)·8THF (**137**) containing di- and tripotassiumsumanenide (reproduced with permission from [68]. Copyright 2015 American Chemical Society).

The cyclopentadienyl (Cp) ligand has its own identity in the field of organometallic chemistry as a plethora of transition metal complexes contain this moiety in their structures. In contrast, not much is reported related to the η^5^-coordinated alkali metal complexes though the first report came around 120 years ago. To this context, in 2015, Hirao and Petrukhina’s group have reported the first alkali metal–sumanene complex K_7_(C_21_H_10_^2−^)_2_(C_21_H_9_^3−^)·8THF (**137)** having di- and tripotassiumsumanenide [68]. As can be inspected from Scheme 36, this complex was prepared by treating the sumanene molecule with an excess of potassium metal. After stirring the reaction for 56 h, the suspension was filtered off and the filtrate was layered in hexanes at 10 °C to yield the beautiful dark-red blocks of the self-assembly. This was confirmed by single crystal structure and it was noticed that all 15 carbon atoms of the three Cp-types of the ring were interacted with the metal atoms and the six K atoms were sandwiched between the convex faces of two sumanenyl trianions.

**Scheme 37:**
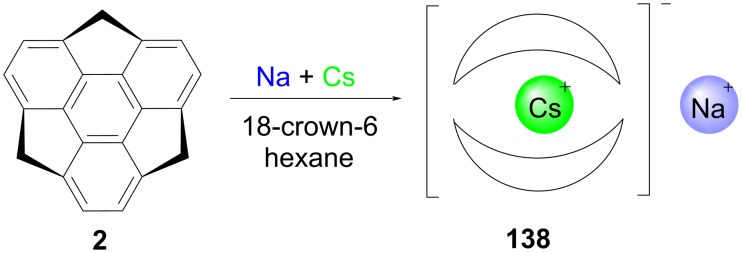
The encapsulation of a Cs^+^ ion between two sumanenyl anions.

On the other hand, two years later to this report, in a really dazzling manner, the groups of Rogachev, Hirao, and Petrukhina reported a novel organometallic sandwich supramolecular complex [Na^+^(18-crown-6)(THF)_2_][Cs(C_2_1H_11_^‒^)_2_]^‒^ (**138**) encapsulating the cesium cation between the sumanenyl anions in a concave manner [69]. To this context, they first prepared a monoanion of sumanene **2** by reacting it with sodium in THF at room temperature and after stirring for 2 hours the cesium was added and stirred for another 8 hours. The reaction mixture was then filtered off and the filtrate was layered with 18-crown-6 in hexane at 10 °C for two days to afford the required complex whose structure was confirmed by X-ray crystallography (Scheme 37).

### 3 Synthesis of heterosumanene derivatives

Doping the backbone of buckybowls such as sumanene and corannulene with heteroatoms (N, P, O, S, Si, and Te) is a promising tactic to modulate the geometrical structure (e.g., bowl depth and bowl-to-bowl inversion energy) and also the physiochemical properties. Therefore, after synthesizing these molecules, scientists turned their attention towards the construction of heterobuckybowls, as heteroatoms could tune the electronic properties of these molecules. Notably, it has been found that the introduction of heteroatom(s) in the periphery of these architectures generally decrease the depth of bowl, may be because of an increase in the carbon–heteroatom bond length. For this reason, these heterobuckybowls possess less strained energy due to shallower bowl structure as compared to the pristine buckybowls containing all-carbon atoms in their frameworks. In sharp contrast, deeper bowl structure (1.30 Å) was observed in the case of triazasumanene as compared to the pristine sumanene (1.11Å) since the C–N bond length (1.47 Å) is shorter compared to the C–C bond (1.54 Å). Due to this, triazasumanene hold high strain energy, hence difficulty in its synthesis and also high reactivity of nitrogen present in it. To date, in most of the heterosumanene substitution has been reported at the benzylic positions except in the case of triazasumanene. In this section we will discuss the developments in heterosumanenes.

#### 3.1 Synthesis of sulfur-doped sumanenes

**Scheme 38:**
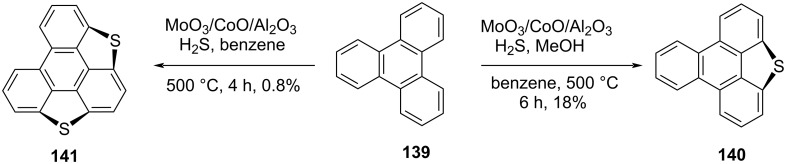
Synthesis of monothiasumanene **140** and dithiasumanene **141** from **139**.

**Scheme 39:**
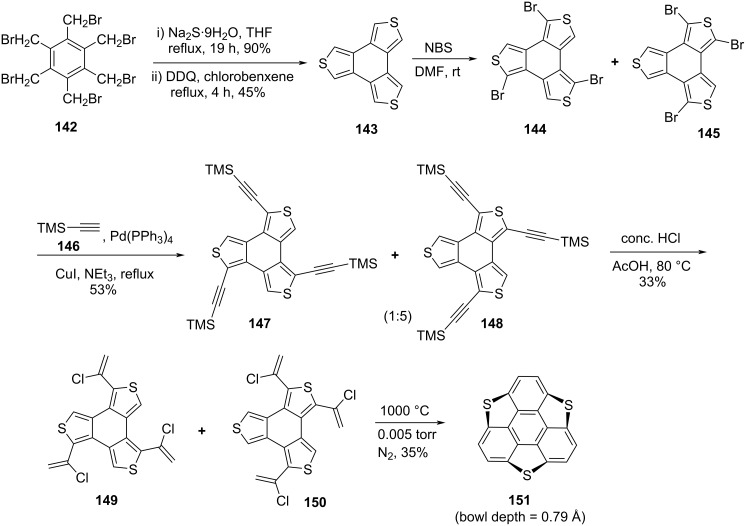
Synthesis of trithiasumanene **151** by Otsubo and his co-workers.

Although, the interest in the sumanene chemistry arise in 1993 when for the first time Mehta and co-workers reported the unsuccessful attempt towards the construction of this beautifully simple yet much valuable π-conjugated buckybowl. Four years later, McGlinchey’s group has tried to synthesize it by means of an organometallic precursor but they also did not make available the breakthrough for the research community [70–71]. On the other hand, four years before to the Mehta’s report, Klemm and co-workers attempted to synthesize trithiasumanene **151** [72] from triphenylene **139** but they could obtain merely mono- and di-bridged systems and no required product was observed (Scheme 38) [73]. However, the group of Otsubo in 1999 provides the breakthrough by successfully synthesizing the first member of the sumanene family, namely trithiasumanene **151** through tribenzannulation of benzotrithiophene **143** by means of a flash-vacuum pyrolysis process as a vital transformation (Scheme 39) [72,74]. As can be observed from an inspection of Scheme 39, their journey for its synthesis commenced from easily assessable 1,2,3,4,5,6-hexakis(bromomethyl)benzene (**142**) to generate benzotrithiophene **143** using sodium sulfide followed by DDQ oxidation. Having benzotrithiophene **143** in hand, it was then subjected to bromination using NBS in DMF to produce two isomeric tribromo derivatives **144** and **145** which were subsequently transformed into the trimethylsilylethynyl derivatives **147** and **148** by employing the Sonogashira coupling reaction. Next, compounds **147** and **148** were reacted with HCl in CH_3_COOH to provide the tris(chlorovinyl) derivatives **149** and **150** which were then subjected to flash vacuum pyrolysis to afford the desired trithiasumanene **151** (Scheme 39).

**Scheme 40:**
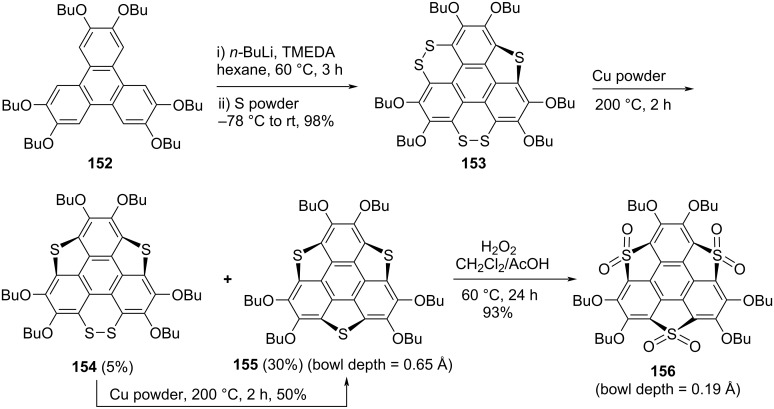
Synthesis of trithiasumanene derivatives **155** and **156**.

On the other hand, Shao and his co-workers synthesized trichalcogenasumanene **155** and tris(*S*,*S*‐dioxides)trithiasumanene **156** by virtue of non-pyrolytic approach as displayed in Scheme 40 [23,75–77]. The sumanene derivative **155** was obtained in two steps first by treating the commercially available triphenylene derivative **152** with *n*-BuLi in the presence of tetramethylethylenediamine (TMEDA) to produce the hexalithiated intermediate which on further treatment with sulfur powder generated the compound **153** having 1,2-dithiin rings as well as one thiophene ring. Later, desulfurizion was accomplished by copper nanopowder to provide the ring-contracted desired product **155** in 30% yield along with a minor amount of **154** which was further converted into the required product under similar reaction conditions (Scheme 40). Moreover, they regioselectively converted **155** into the sumanene-based trisulfone derivative **156** in the presence of hydrogen peroxide in AcOH as detailed in the Scheme 40.

**Scheme 41:**
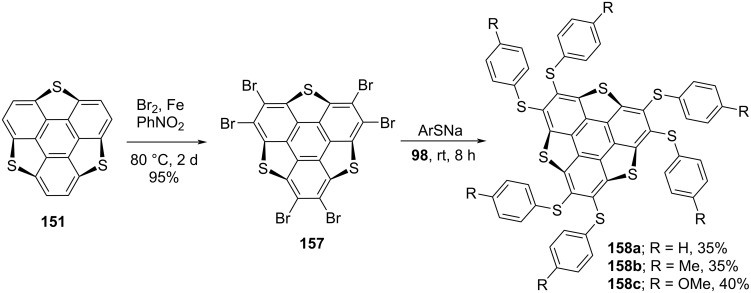
Synthetic route towards hexathiolated trithiasumanenes **158**.

Liu et al. has revealed the functionalization of a sulfur-doped sumanene by means of perbromination followed by nucleophilic substitution as depicted in Scheme 41 [78]. They first performed the hexabromination using Br_2_ and iron powder in PhNO_2_. The brominated derivative **157** was then converted to the hexathiolated trithiasumanenes **158a–c** by substitution reaction (Scheme 41). The structures of these functionalized heterosumanenes were confirmed by spectroscopy as well as crystallography means.

#### 3.2 Synthesis of triselenasumanene

**Scheme 42:**
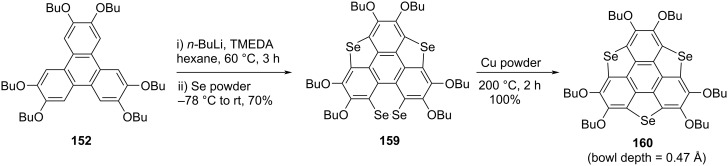
Synthesis of triselenasumanene **160** by Shao and teammates.

The first synthesis of triselenasumanene **160** was also performed in the laboratory of Shao’s group as depicted in Scheme 42 [23]. The sumanene derivative **160** was prepared from the triphenylene system **152** by treating it with *n*-BuLi in the presence of TMEDA followed by quenching the hexaanionic species with selenium powder to afford **159** containing one 1,2-diselenin ring and two selenophene rings. Compound **159** was later subjected to deselenation in the presence of copper nanopowder (80–100 nm grain size) to furnish the expected compound **160** in quantitative yield (Scheme 42).

#### 3.3 Preparation of tritellurasumanene derivatives

**Scheme 43:**
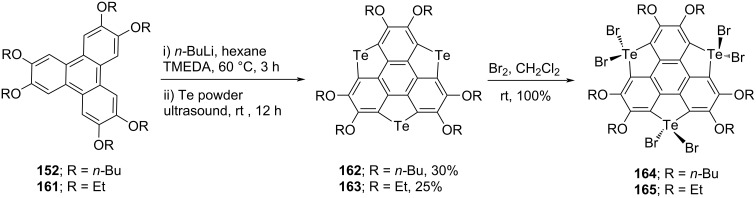
Synthesis of tritellurasumanene derivatives from triphenylene skeletons.

On the other occasion, tritellurasumanene derivatives **162**–**165** have been prepared by Shao’s team starting from triphenylene skeletons by means of an ultrasound-assisted one-pot procedure (Scheme 43) [79–80]. As can be noted from Scheme 43, the hexalithiation of **152** was accomplished using butyllithium in the presence of TMEDA which on further treatment with tellurium powder afforded the desired product **162** in 30% yield with 60% starting material recovery. Along similar lines, they also assembled compound **163** in 25% yield as displayed in Scheme 43. Furthermore, when they subjected the tritellurasumanenes **162** and **163** with Br_2_ in CH_2_Cl_2_, they obtained the tris(*Te,Te*-dibromo)tritellurasumanenes **164** and **165** as covalent adducts in quantitative yield (Scheme 43).

#### 3.4 Preparation of sulfur, selenium and tellurium-doped sumanenes

**Scheme 44:**
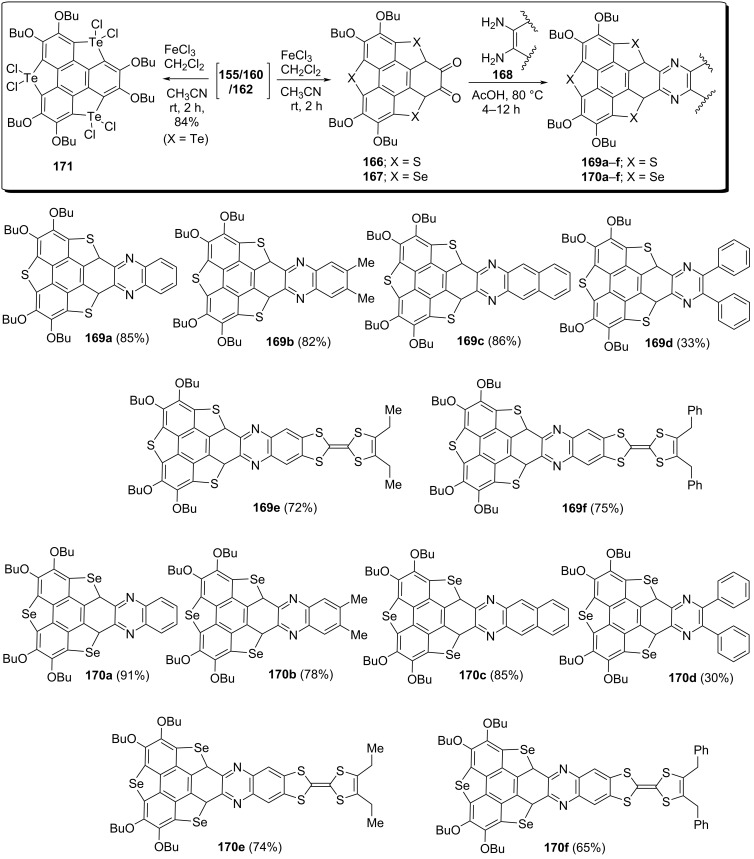
Synthesis of pyrazine-fused sumanene architectures through condensation reaction.

The pyrazine-fused trichalcogenasumanenes have been assembled via the transformation of 1,2-dibutoxybenzene unit into the corresponding *ortho*-quinones by means of FeCl_3_ in CH_2_Cl_2_/MeCN at room temperature (Scheme 44) [79–81]. Interestingly, it was observed that 1,2-dibutoxy groups could be selectively transformed to the *ortho*-quinones in the presence of FeCl_3_. From the experimentations, they noticed that heteroatoms also play a significant role in this reaction for instance with sulfur and selenium-doped systems, *ortho*-quinones **166** and **167** were obtained (Scheme 44). In sharp contrast, the tellurium-doped sumanene provided the covalent adduct **171** with Cl atoms attached onto the Te atoms, confirmed by an X-ray analysis (Scheme 44). Having the quinone derivatives **166** and **167** in hands, they were next subjected to the condensation reaction with a variety of aryl-1,2-diamines **168** in the presence of AcOH to generate the corresponding pyrazine-fused sumanene networks **169a–f** and **170a–f** in low-to-good yields (Scheme 44).

**Scheme 45:**
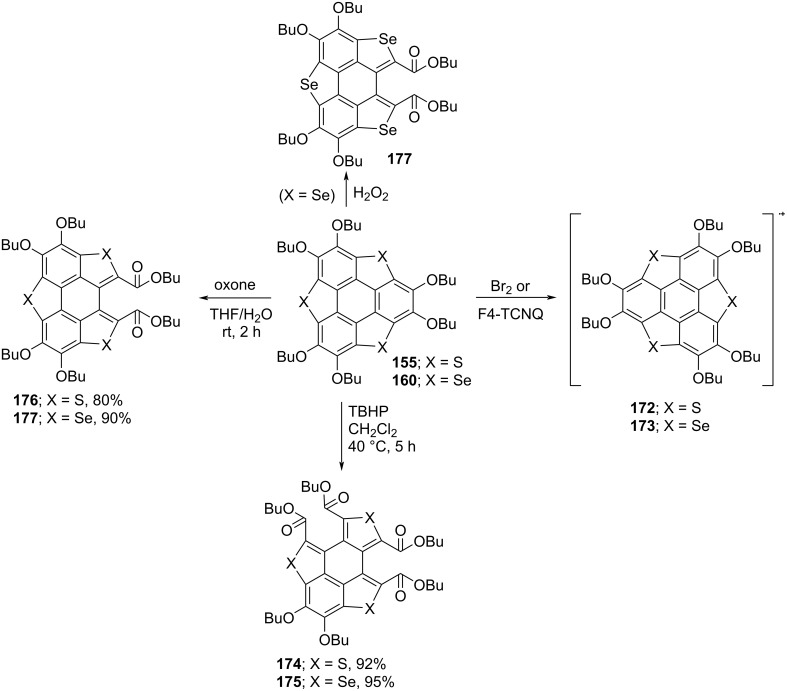
Treatment of the trichalcogenasumanenes with diverse oxidative reagents.

**Scheme 46:**
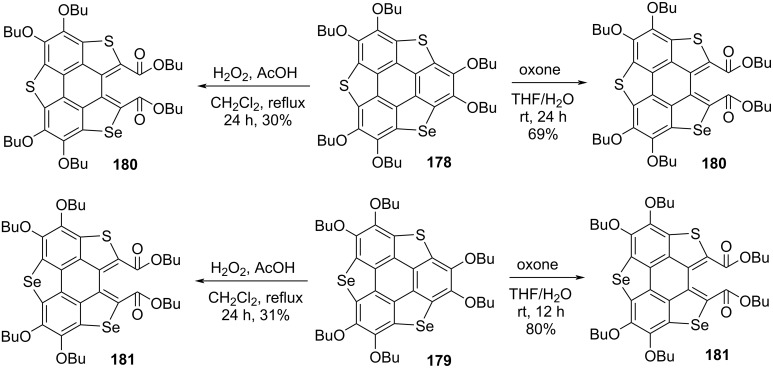
Ring-opening reaction with H_2_O_2_ and oxone of heterasumanenes **178** and **179**.

**Scheme 47:**
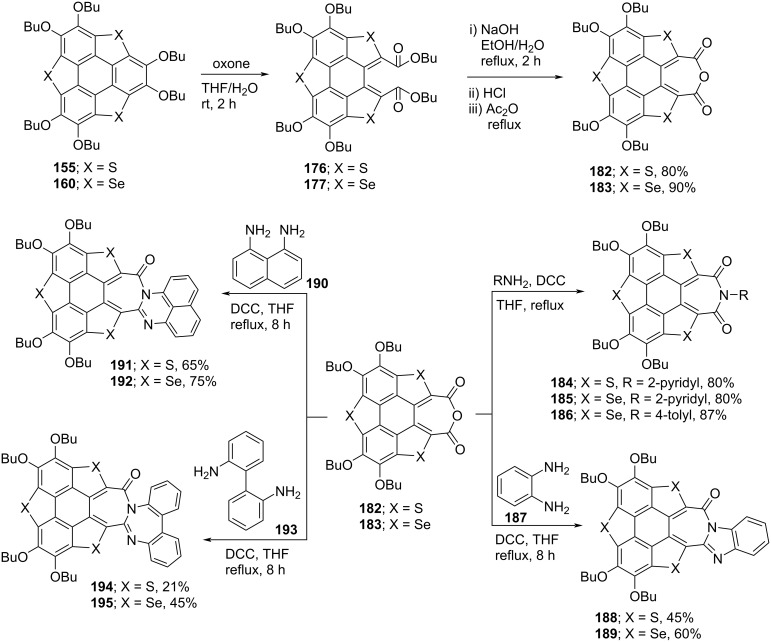
Synthesis of polycyclic compounds from sumanene derivatives.

**Scheme 48:**
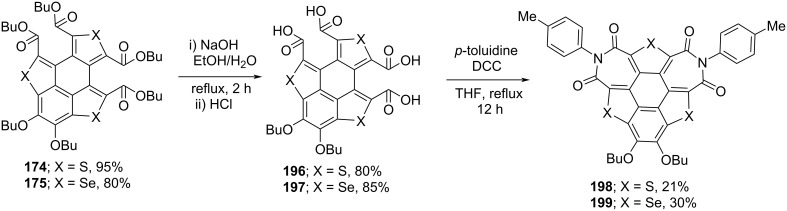
Synthesis of diimide-based heterocycles reported by Shao’s and co-workers.

As can be inspected from Scheme 45, oxidation of **155** and **160** with both oxone (potassium peroxymonosulfate) and H_2_O_2_ afforded the one-ring-opened product **177**. Interestingly, when these compounds were treated with *tert*-butyl hydroperoxide (TBHP), two flanking benzene rings were found to be cleaved to afford **174** and **175** in impressive yields (Scheme 45) [82–84]. Moreover, they observed radical cations **172** and **173** formation when the same compounds were reacted with Br_2_ or 2,3,5,6-tetrafluoro-7,7,8,8-tetracyanoquinodimethane (F_4_-TCNQ). On the other hand, when compounds **178** and **179** were reacted with oxidizing agents such as oxone and H_2_O_2_, selectively one benzene ring-cleaved products **180** and **181** were isolated (Scheme 46) [85]. Having the ring-opened products in hands, they were then subjected to the functional group transformation (molecular-surgery-type functionalization) by treating them with NaOH in EtOH/H_2_O (10:1) under reflux conditions to first convert esters functionality into the corresponding carboxylic acid derivatives. As displayed in Scheme 47, these acid derivatives were then transformed into acid anhydrides which on further treatment with different aromatic amines afforded a variety of polyheterocylic compounds [82,86]. Furthermore, Shao’s group has also assembled the diimide-based heterocycles as depicted in Scheme 48 [83].

**Scheme 49:**
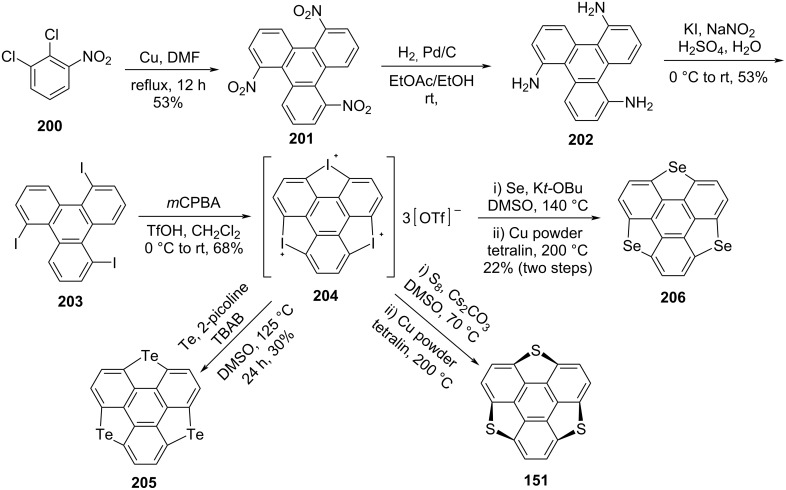
Synthesis of pristine trichalcogenasumanenes, **151**, **205**, and **206**.

**Scheme 50:**
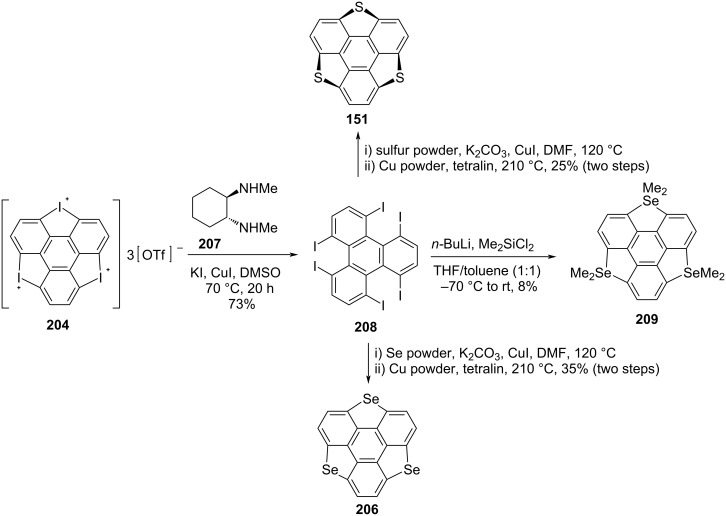
Synthesis of trichalcogenasumanenes via hexaiodotriphenylene precursor **208**.

Quite recently, Tan et al. has disclosed a simple yet effective strategy for scalable synthesis of pristine sulfur, selenium and tellurium-doped sumanenes starting from 1,2-dichloro-3-nitrobenzene **200** by employing the Ullmann coupling and Sandmeyer reaction as key transformations (Scheme 49) [18,87–88]. The target was achieved starting from a three-fold Ullmann coupling reaction of **200** to produce the *C*_3_-symmetric trinitrotriphenylene **201**. Having compound **201** in hand, the nitro groups were further converted into the corresponding amino groups via reduction in the presence of Pd/C under hydrogen gas atmosphere to furnish compound **202** in almost quantitative yield. Next, compound **202** was treated with KI/NaNO_2_ under Sandmeyer reaction conditions to afford the triiodo compound **203** which on subsequent reaction with *m*CPBA and triflic acid (TfOH) provided the iodine-doped sumanene **204** in 68% yield. Finally, compound **204** was converted to the required tritellurasumanene **205** through the reaction with Te powder in the presence of tetrabutylammonium bromide (TBAB) and 2-picoline in DMSO as shown in Scheme 49. Interestingly, as can be pointed out from Scheme 49, pristine trithiasumanene **151** and triselenasumanene **206** were also obtained from the common iodine-doped sumanene building block **204**. Furthermore, the precursor **204** was converted to the 1,4,5,8,9,12-hexaiodotriphenylene **208** via ring-opening with KI in the presence of a copper/diamine catalyst (Scheme 50). Gratefully, compound **208** was also used as building block for the construction of heterasumaneness **151**, **206** and **209** as depicted in Scheme 50.

#### 3.5 Synthesis of trisilasumanene systems

**Scheme 51:**
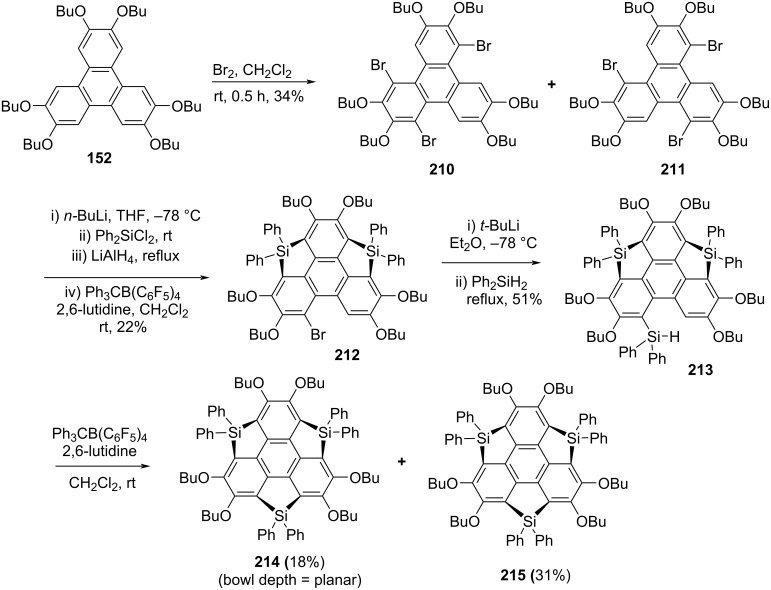
Synthesis of trisilasumanenes **214** and **215**.

The synthesis of trisilasumanene **214** from triphenylene derivative **152** has been achieved by Furukawa, Kobayashi and Kawashima using the intramolecular sila-Friedel–Crafts reaction as a crucial step (Scheme 51) [89–92]. In this context, they embarked with the bromination of triphenylene derivative **152** using bromine in CH_2_Cl_2_ to produce two isomeric tribromotriphenylene derivatives **210** and **211** as depicted in Scheme 51. The doubly cyclized monobromo derivative **212** was obtained in two steps from a mixture of tribromotriphenylenes, first by installing two Ph_2_HSi groups into it through the lithiation using butyllithium and subsequent addition of Ph_2_SiCl_2_ followed by reduction with LiAlH_4_. Next, the compounds containing Ph_2_Si groups were treated with Ph_3_CB(C_6_F_5_)_4_ to generate silicenium ionic intermediates which on further two-fold intramolecular sila-Friedel–Crafts cyclization reaction furnished compound **212**. Lastly, by repeating the same steps under similar reaction conditions, the desired trisilasumanene **214** was obtained along with a desilylated product **215** (Scheme 51).

**Scheme 52:**
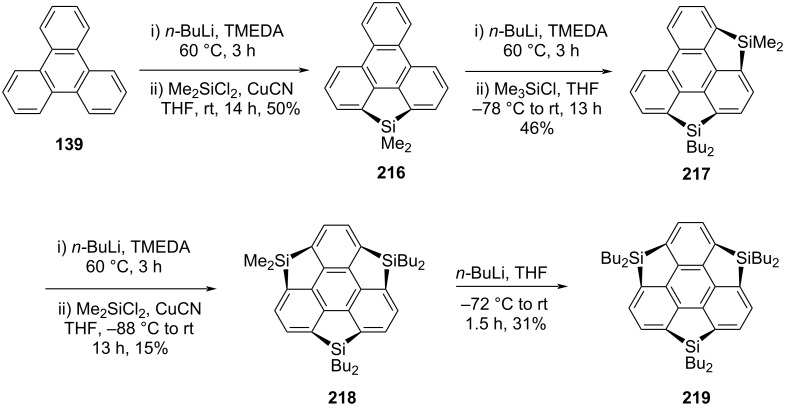
Synthesis of trisilasumanene derivatives **218** and **219**.

On the other hand, two years later of the Kawashima’s report, Saito’s revealed the synthesis of novel trisilasumanenes **218** and **219** as shown in Scheme 52 [93]. Their exploration of these attractive architectures commenced with the formation of dilithiotriphenylene using butyllithium and TMEDA and subsequent reaction with dichlorodimethylsilane to deliver the monocyclized compound **216** in 50% yield (Scheme 52). For the formation of another ring, compound **216** was further treated with *n*-BuLi in the presence of TMEDA to generate the dianion which on subsequent quenching with Me_3_SiCl afforded compound **217** in 46% yield. The final step for the construction of the first trisilasumanene **218** containing no functional groups on the benzene ring system involve the bridging of the remaining bay positions of **217** which was achieved by treating it with *n*-BuLi followed by the addition of Me_2_SiCl_2_ to provide title compound **218** in low yield. Furthermore, by reacting compound **218** with butyllithium yielded the required novel *C*_3_-symmetric hexabutyltrisilasumanene **219** in 31% yield (Scheme 52).

#### 3.6 Construction of novel trigermasumanene

**Scheme 53:**
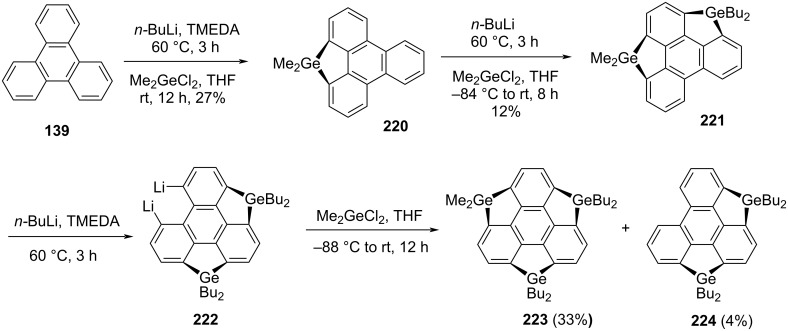
Synthesis of novel trigermasumanene derivative **223**.

One year later, Saito and co-workers have prepared the trigermasumanene **223** having no substituents on the benzene rings through the repetitive lithiation starting from the triphenylene system **139** followed by the insertion of germanium functionalities as displayed in Scheme 53 [94]. As discussed above for the construction of trisilasumanene **218**, herein they have used a similar strategy involving identical steps to achieve the desired target **223** as depicted in Scheme 53.

#### 3.7 An attempt towards the synthesis of tristannasumanene

**Scheme 54:**
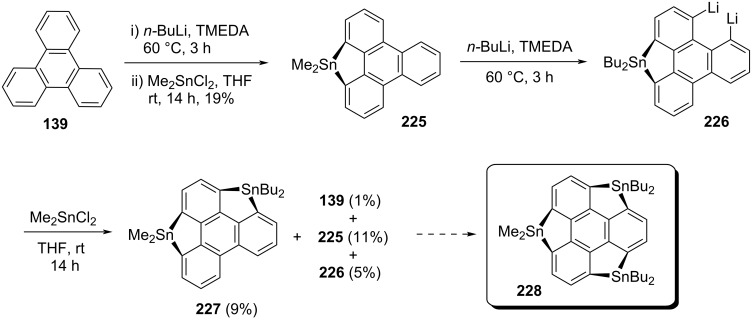
An attempt towards the synthesis of tristannasumanene derivative **228**.

To further advance the chemistry of heterasumanenes, Saito’s group has also put their unsuccessful effort for the preparation of tristannasumanene by employing the similar tactic as discussed above for trisila- and trigermasumanene derivatives [94]. As can be inspected from Scheme 54, the monocyclic compound **225** was achieved in 19% yield which on further treatment with butyllithium and TMEDA followed by quenching with Me_2_SnCl_2_ provided **227** in just 9% yield along with **139**, **225**, **226** as shown in Scheme 54. In this procedure, they noticed the transmetalation between tin and lithium atoms. Therefore, because of the cleavage of the Sn–C bonds competitively during the lithiation steps at the bay regions, it’s difficult to obtain the final sumanene derivative **228**. Hence, they suggested for an alternative route to assemble the tristannasumanene derivative.

#### 3.8 Synthesis of phosphorous-doped sumanene derivatives

**Scheme 55:**
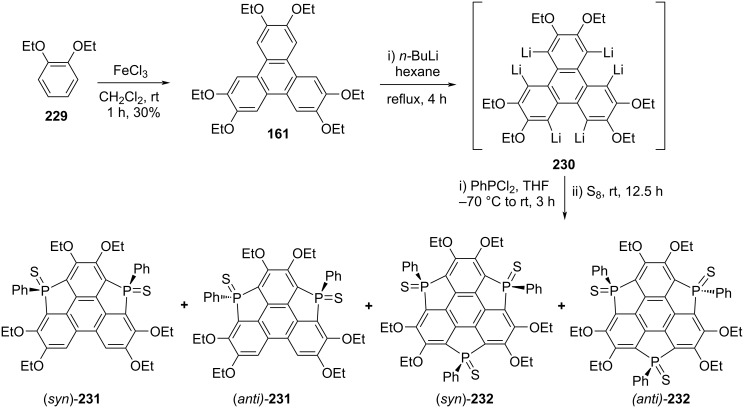
Synthesis of triphosphasumanene trisulfide **232** from commercially available **229**.

In 2017, the groups of Furukawa, Tada, Fujii and Saito have reported the synthesis of triphosphasumanene trisulfide **232** from 2,3,6,7,10,11-hexaethoxytriphenylene (**161**), prepared by oxidative coupling of **229** (Scheme 55) [95]. The hexalithiated intermediate **230** was obtained by treating the compound **161** with excess of *n*-BuLi under hexane reflux conditions. The intermediated **230** was then reacted with dichlorophenylphosphine and subsequent addition of elemental sulfur to furnish the compounds (*syn*)-**232** and (*anti*)-**232** along with triphenylenodiphosphole disulfides (*syn*)-**231** and (*anti*)-**231** (Scheme 55).

#### 3.9 Heterasumanenes containing different heteroatom functionalities

**Scheme 56:**
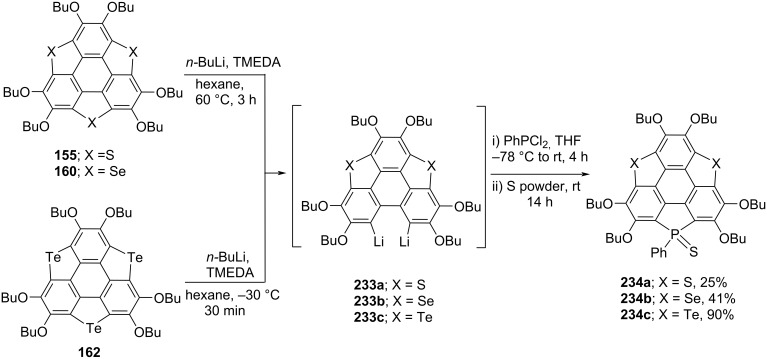
The doping of sumanene derivatives with chalcogens (S, Se, Te) and phosphorus.

To advance the heterasumanenes chemistry possessing value-added functionalities, doping sumanene with both chalcogen and phosphorus seems to be much promising. To this context, in 2019, Wang et al. have prepared the heterasumanenes **234a–c** having chalcogens (S, Se, and Te) and phosphorus atoms at the benzylic positions of the sumanene derivatives starting from trichalcogenasumanenes in a single step (Scheme 56) [96]. In this report, they skillfully opened-up one of the rings of trichalcogenasumanenes by means of butyllithium to produce the dilithiated intermediates **233a–c** which on subsequent treatment with dichlorophenylphosphine (PhPCl_2_) followed by the addition elemental sulfur produced the required sumanene derivatives **234a–c** in moderate-to-excellent yields. Surprisingly, it was observed that when compound **162** reacted with butyllithium at 60 °C, two of the rings were found to be opened. Therefore, they carried out the reaction at lower temperature to open only one of the rings to generate **233c** which was further transformed into the heterosumanene **234c** as can be inspected from Scheme 56.

**Scheme 57:**
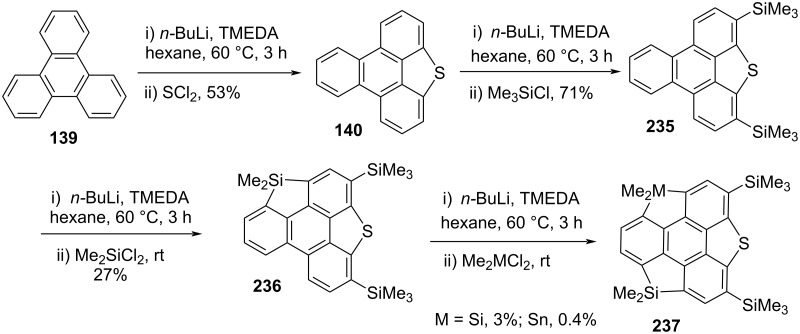
Synthesis of heterasumanene containing three different heteroatoms.

In another experiment, Saito and his co-workers disclosed heterasumanenes **237** possessing three dissimilar heteroatom functionalities as shown in Scheme 57 [97–98]. Their journey towards this goal stems from the triphenylene skeleton **139** to produce the triphenyleno[1,12-*bcd*]thiophene **140** which on further reaction with butyllithium and Me_3_SiCl generated the bis(trimethylsilyl) derivative **235**. Having compound **235** in hand, it was then transformed into **236** by lithiation and subsequent quenching with Me_2_SiCl_2_. The final step was to arch the residual bay positions by virtue of heteroatom involving the same sequence of reactions under similar reaction conditions (Scheme 57).

**Scheme 58:**
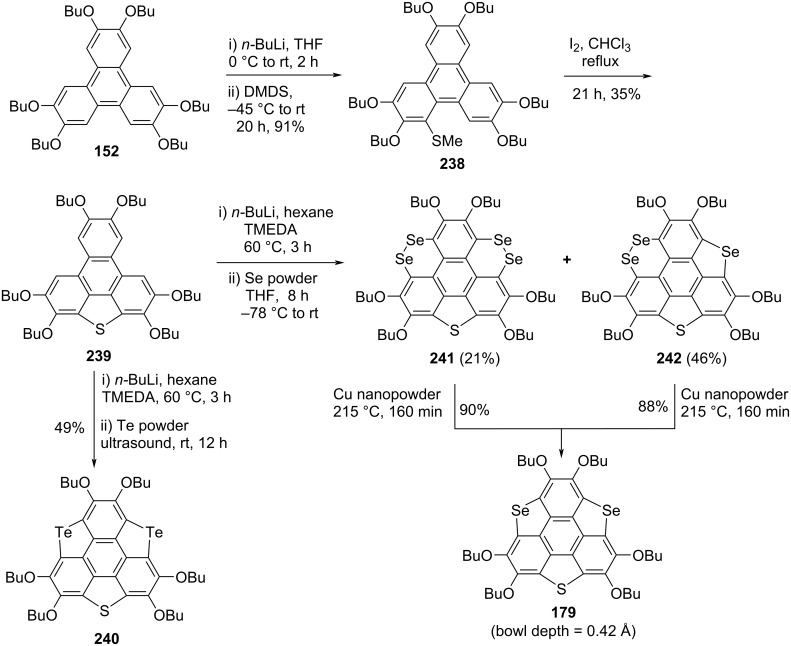
Synthesis of trichalcogenasumanene derivatives **240** and **179**.

**Scheme 59:**
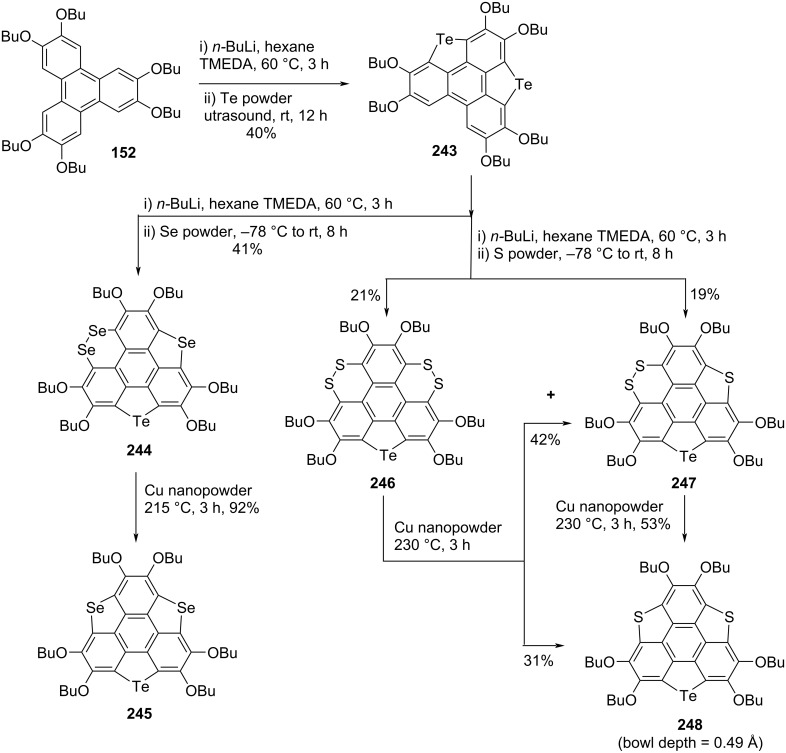
Preparation of trichalcogenasumanenes **245** and **248**.

**Scheme 60:**
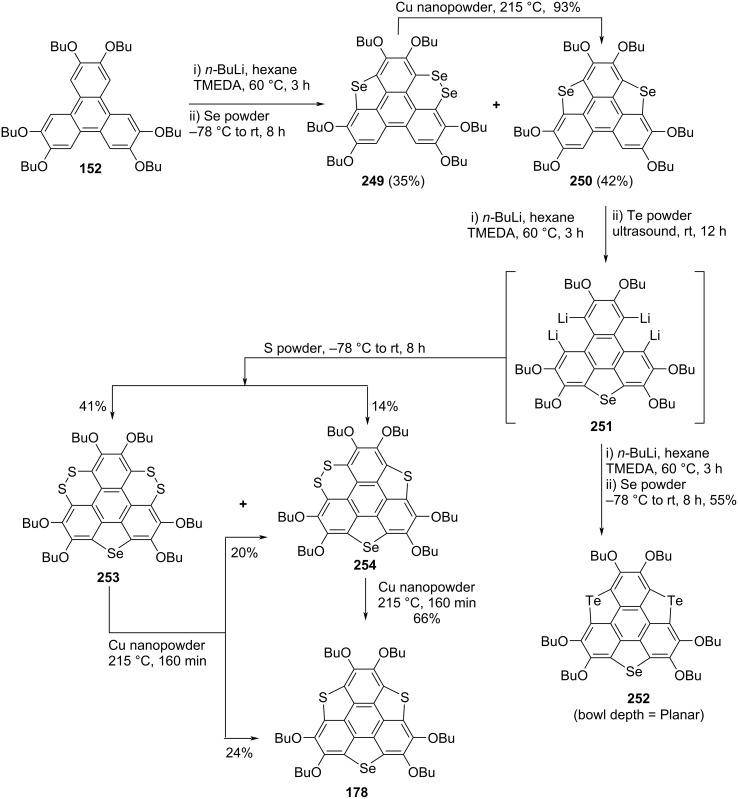
Design and synthesis of trichalcogenasumanene derivatives **252** and **178**.

Since trichalcogenasumanene and their congeners are gifted with rich and fascinating chemistry, as regioselective functionalization of these attractive molecules could be easily operated. The chalcogen atom(s) containing molecules particularly having S, Se, and Te atoms in their structures play a crucial role in determining the optical properties, molecular geometry (bowl-to-planar), and chemical reactivities. Having this in mind, very recently a series of trichalcogenasumanene derivatives by means of benzylic carbon replacement with two kinds of chalcogen atoms has been exposed from Shao’s laboratory (Schemes 58–60) [85]. The synthesis of sumanene derivatives **240** and **179** began with the same starting material 2,3,6,7,10,11-hexabutoxytriphenylene (HBT) **152** by treating it with butyllithium and dimethyl disulfide (DMDS) to produce the triphenylene derivative **238** in excellent yield. The compound **238** was further converted into the building block **239** by reacting it with iodine in refluxing CHCl_3_ which on subsequent treatment with *n*-BuLi followed by the addition of tellurium powder provided the required compound **240**. Alternatively, after lithiation with butyllithium and subsequent addition of selenium powder, compound **239** provided **241** and **242** in 21% and 46% yields, respectively. These compounds **241** and **242** were then converted into the desired sumanene derivative **179** upon treatment with copper nanopowder as shown in Scheme 58. Furthermore, as can be seen from an inspection of Scheme 59 and Scheme 60, these authors have used almost similar repetitive steps to assemble the other sumanene derivative such as **245**, **248**, **252**, and **178** starting from HBT (**152**).

**Scheme 61:**
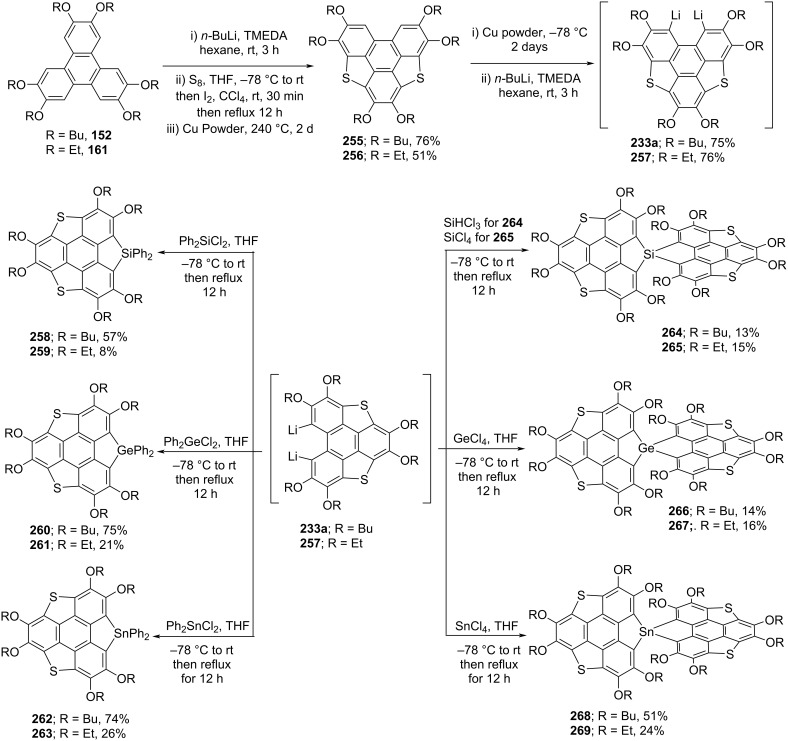
Synthesis of spirosumanenes **264–269** and non-spiroheterasumanenes **258–263**.

To advance the chemical space of heterasumanenes, in 2018, the groups of Furukawa and Saito have reported the synthesis of spiro-heterasumanenes possessing the elements of group 14 (Si, Ge, and Sn) as bridging atoms (Scheme 61) [99]. Towards this mission, they started with the lithiation of hexasubstituted triphenylene derivatives **152** and **161** in the presence of butyllithium and subsequently adding the sulfur/iodine to generate the compounds **255** and **256** in good yields. The compounds **255** and **256** were then treated with copper powder for desulfurization and further subjected to lithiation using butyllithium to generate dilithiated intermediates **233a** and **257**. Having these valuable intermediates **233a** and **257** in hands, they were then treated with heteroatom reagents (MCl_4_, M = Si, Ge, Sn or SiHCl_3_) to produce the corresponding spiro-sumanene derivatives **264–269** in moderate yields. Moreover, the non-spiro-type heterasumanenes **258–263** were also assembled by reacting with heteroatom reagents (Ph_2_XCl_2_, X = Si, Ge, Sn) in low-to-good yield as shown in Scheme 61.

**Scheme 62:**
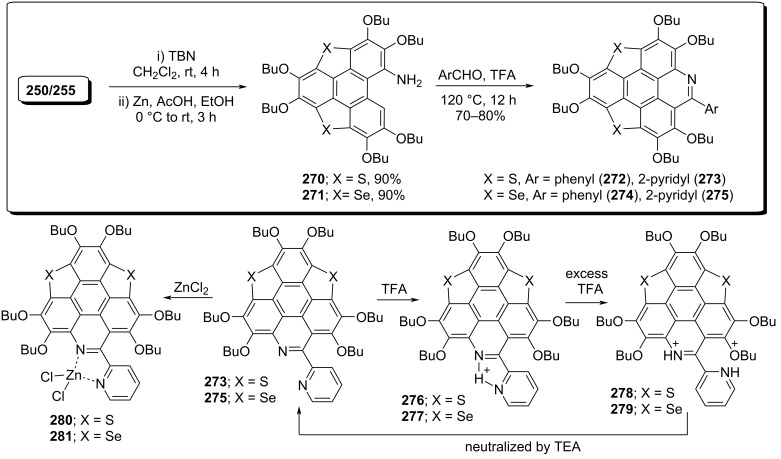
Synthesis of sumanene-type hetero polycyclic compounds.

To further exploit the chemistry of sumanenes, Shao and co-workers have constructed several sumanene-type hetero-polycycles and they further noticed that the planar **273** and **275** could be easily transformed into the bowl-shaped molecules via the chelatation of Zn^2+^ ion with the bipyridyl unit at room temperature through the formation of a five-membered ring system (Scheme 62) [100]. Noticeably, they observed that this coordinated system displays totally different optical properties compared to the parent molecules. To achieve the target, they commenced with the compounds **250/255** by reacting them with *tert*-butyl nitrite (TBN) to provide the corresponding mononitro compounds in good yields. Having these nitro compounds in hands, they were next subjected to the reduction using Zn/AcOH to furnish the amino group-containing compounds **270** and **271**. These compounds were then treated with pyridine-2-aldehyde and benzaldehyde in a Pictet–Spengler fashion to yield the pyridine-based sumanene-type molecules **272–275**. Interestingly, they noticed that when the compounds **273** and **275** were treated with trifluoroacetic acid (TFA), the protonation occurs only at the hanging pyridine ring system. Whereas in excess of TFA both the pyridine rings of the bipyridyl system got protonated which on further neutralization with triethylamine (TEA) produced the deprotonated compounds back (Scheme 62). Moreover, when these compounds were titrated with ZnCl_2_, the coordination was occurred with zinc cations forming the five-membered chelate as displayed in Scheme 62.

#### 3.10 Synthesis of chiral nitrogen-doped sumanene and its congeners

**Scheme 63:**
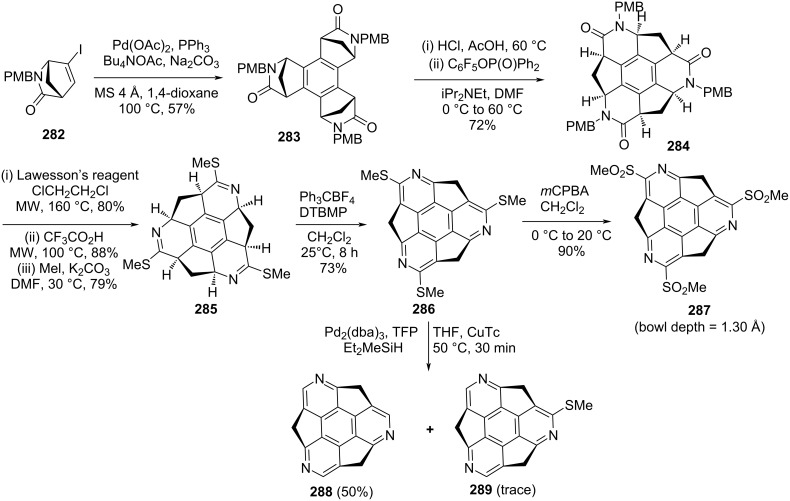
Synthesis of triazasumanenes **288** and its sulfone congener **287**.

To explore the diversity of heteroatom-doped sumanene derivatives, Tan, Higashibayashi, Karanjit, and Sakurai reported the first enantioselective synthesis of triazasumanenes, being more curved and displaying a larger bowl depth than pristine sumanene (**2**), confirmed by X-ray analysis (Scheme 63) [20,101]. The synthetic strategy for triazasumanenes began with the palladium-catalyzed cyclotrimerization of enantiopure (1*S*,4*R*)-**282** to yield the *C*_3_-symmetric compound **283** which on hydrolysis and further condensation afforded the non-conjugated lactam **284** (Scheme 63). Since several unsuccessful experiments were performed to directly convert **284** into the desired aromatic bowl-shaped triazasumanene. Therefore, an alternative route was chosen which involve first conversion of the lactam **284** to the thioimidate **285** using Lawesson’s reagent, and then deprotection of PMB followed by methylation using MeI/K_2_CO_3_. Furthermore, dehydrogenation of **285** was successfully carried out using Ph_3_CBF_4_ and 2,6-di-*tert*-butylpyridine (DTBMP) to afford the aromatized compound **286**. Since a single crystal of the heterosumanene **286** could not be obtained, they transformed it into the corresponding sulfone derivative **287** whose X-ray study was successful carried out. On the other hand, Sakurai and co-workers have also reported the pristine triazasumanene **288** by performing the desulfurization using poly(methylhydrosiloxane) (PMHS) or Et_2_MeSiH in the presence of Pd_2_(dba)_3_/tris(2-furyl)phosphine (TFP) along with copper(I) thiophene-2-carboxylate (CuTC) (Scheme 63) [20].

**Scheme 64:**
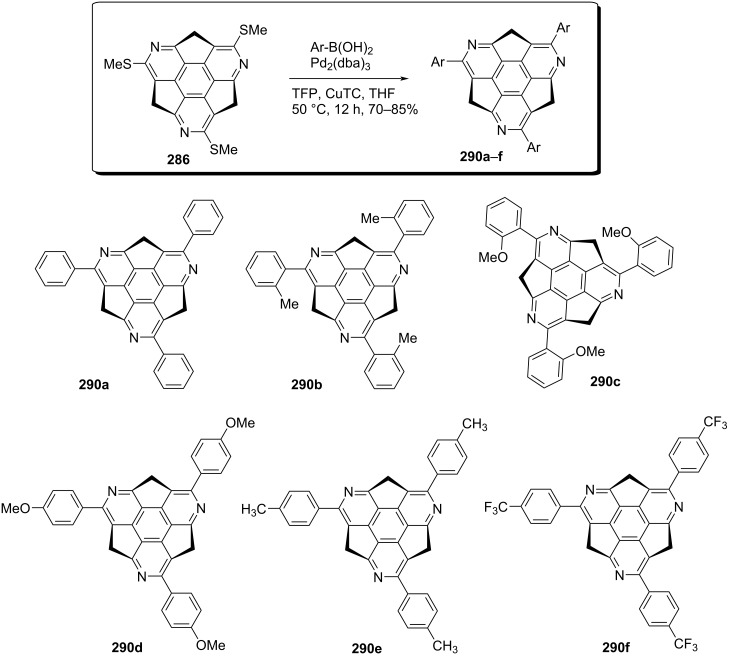
Synthesis of *C*_3_-symmetric chiral triaryltriazasumanenes via cross-coupling reaction.

In another paper, Sakurai’s group has also reported a series of *C*_3_-symmetric chiral triaryltriazasumanene derivatives **290a–f** from chiral tris(methylthio)triazasumanene **286** using diverse boronic acids employing a palladium-catalyzed cross-coupling reaction (Scheme 64) [102]. The coupling reaction was performed between different boronic acids and sumanene derivatives **286** using Pd_2_(dba)_3_, CuTC and TFP in THF at 50 °C. They have assembled a variety of triazasumanene derivatives using this wonderful strategy in decent yields (Scheme 64).

### 4 Synthesis of higher-order bowl-shaped sumanene molecules

**Scheme 65:**
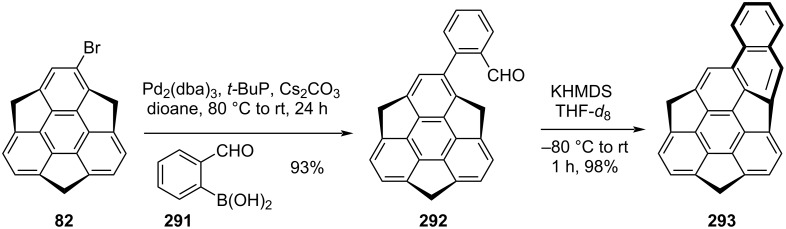
Synthesis of mononaphthosumanene **293** using Suzuki coupling as a key step.

**Scheme 66:**
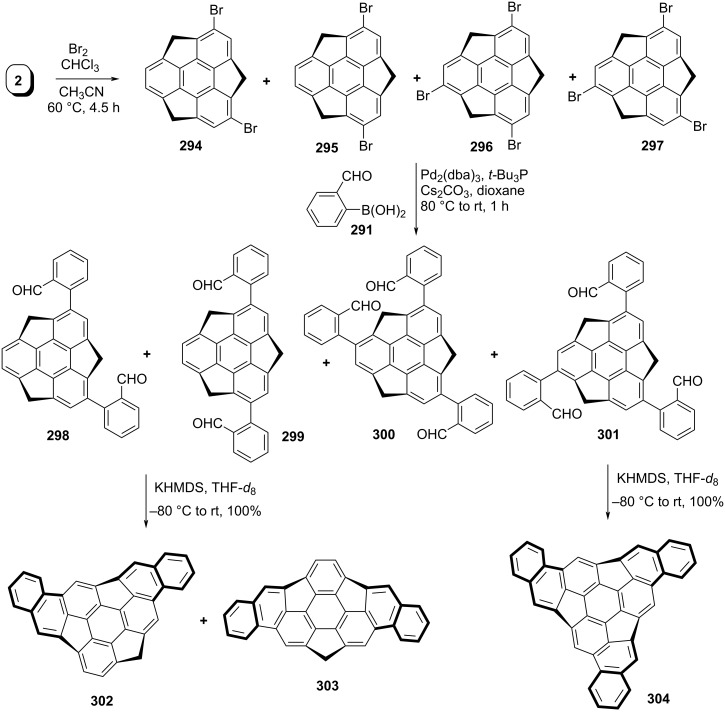
Synthesis of di- and trinaphthosumanene derivatives **302–304**.

In addition to C_60_ and carbon nanotubes (CNTs), the buckybowls are also considered as an interesting class of materials. Although, the chemistry of the corannulene scaffold is highly explored but the sumanene series is still immature due to synthetic difficulties because of the presence of three pentagonal and four hexagonal rings causing high strain. The deeper buckybowls are of significant interest as they possess more similar properties to those of CNTs and fullerenes. These interesting π-bowls are not only expected to be the building block for the construction of fullerene and carbon CNT skeletons but are also useful in liquid crystals and organic semiconductors. The sumanene scaffold is of much interest compared to corannulene not only because it has a large bowl depth but also due to the presence of available benzylic positions for further functionalization. Since these bowl-shaped molecules could also be used for the preparation of fullerenes in a bottom-up approach. Therefore, in recent years, a significant attention of the research community is directed towards the construction of higher-order bowl-shaped architectures. In this context, Hirao and his co-workers reported the synthesis of mono, di- and trinaphthosumanenes using benzannulation reactions (Scheme 65 and Scheme 66) [103]. Towards this mission, the first investigation was carried out by utilizing the benzannulation reaction on momobromosumanene **82** to afford the corresponding mononaphtosumanene **293** by means of a Suzuki-coupling reaction with 2-formylphenylboronic acid **291** followed by an intramolecular condensation reaction to afford the required sumanene derivative **293** in excellent yield (Scheme 65). Furthermore, to obtain the di- and trinaphthosumanenes, they stem from the bromination of pristine sumanene (**2**) in the presence of molecular bromine to generate an inseparable mixture of dibromosumanenes **294** and **295** and tribromosumanenes **296** and **297**. The Pd-catalyzed Suzuki–Miyaura cross-coupling reaction of this mixture with boronic acid **291** provided the corresponding separable compounds **298–301** in overall good yields. The compounds **298** and **299** on two-fold benzannulation in the presence of KHMDS afforded a mixture of **302** and **303** in a 1:1.2 ratio. In contrast, only compound **301** underwent three-fold benzannulation reaction to afford the trinaphthosumanene **304** as depicted in Scheme 66.

**Scheme 67:**
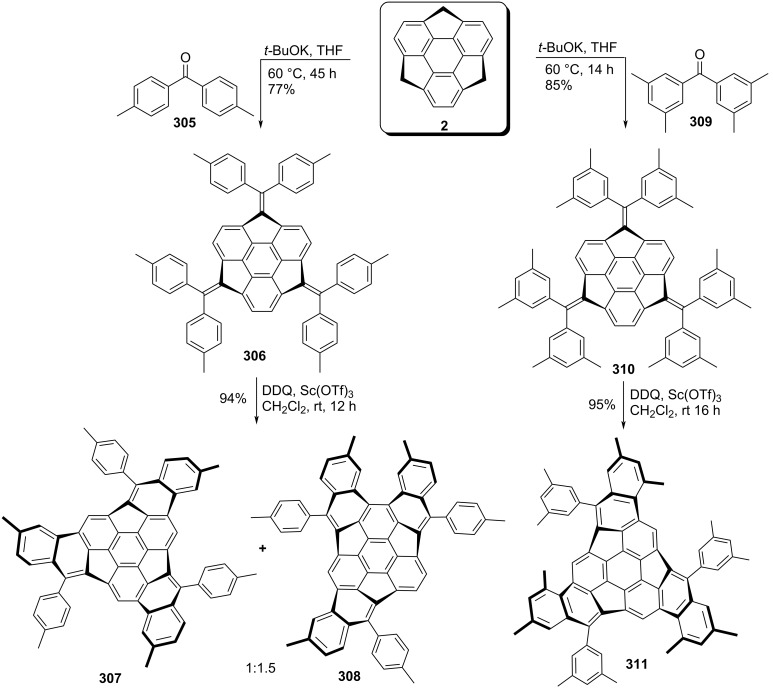
Synthesis of hemifullerene skeletons by Hirao’s group.

On the other hand, in a truly brilliant way, Hirao’s team has reported the synthesis of the hemifullerene skeleton in just two steps starting from sumanene **2** by involving the regioselective intramolecular oxidative cyclization as a critical step (Scheme 67) [104]. To achieve their target, they first performed the condensation reaction of sumanene (**2**) with benzophenone derivatives **305** and **309** utilizing *t*-BuOK to give **306** and **310** in 77% and 85% yields, respectively. The dehydrogenative oxidative cyclization of **306** using DDQ in the presence of Sc(OTf)_3_ afforded the *C*_3_-symmetric molecule **307** along with **308** in 94% yield with 1:1.5 ratio. Whereas compound **310** solely produced the hemifullerene **311** in excellent yield under similar reaction conditions (Scheme 67).

**Scheme 68:**
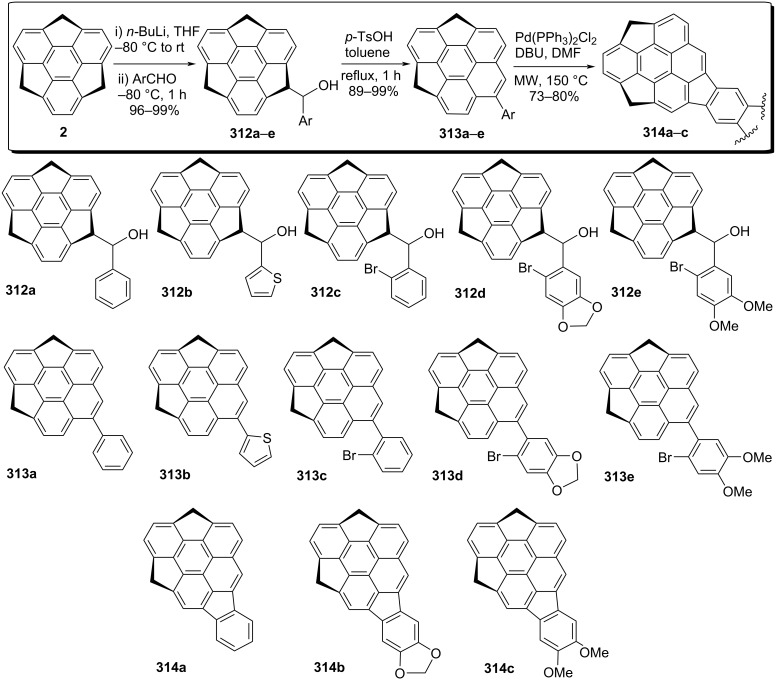
Design and construction of C_70_ fragment from a C_60_ sumanene fragment.

More strikingly, the Sakurai group has reported the synthesis of a buckybowl fragment of C_70_ from a C_60_ sumanene fragment through the ring expansion and annulation reactions in three steps including a Wagner–Meerwein rearrangement to transform the five-membered ring to a six-membered ring as a key transformation (Scheme 68) [105–106]. Their synthetic plan to this goal started with the formation of benzylic carbanion using butyllithium followed by the reaction with a range of aromatic aldehydes to generate the arylsumanyl alcohols **312a–e** in 96–99% yields. The Wagner–Meerwein rearrangement was then performed by treating these alcohols **312a–e** with stoichiometric amounts of *p*-TsOH under toluene reflux conditions to afford the corresponding benzopyrene derivatives **313a–e** in 89–99% yields. Finally, the required C_70_ fragment of buckybowls **314a–c** were achieved through the cyclization reaction of *o*-brominated derivatives **313a–c** using Pd(PPh)_2_Cl_2_ and DBU under microwave reaction conditions as depicted in Scheme 68.

## Conclusion

The discovery of the pristine sumanene inspired a new wave of investigations on heteroatom-doped sumanene derivatives, and significant developments have been made in the past one and half decades. More interestingly, in recent years, the chemistry of sumanene is continuously attracting tremendous interest of the research community because of their outstanding physiochemical properties as well as potential applications spanning from organometallics, organic chemistry to the supramolecular chemistry and materials science. Although, the chemistry of coronnulene systems is already matured but sumanene, one of the most beautiful and fascinating classes of buckybowl architectures is yet to be explored to higher level. Therefore, to provide readers a quick overview of where the field has been, where it stands now, and where it might be going in near future, herein we have comprehensively summarized all the available synthetic strategies towards the construction of sumanene and its congeners since its invention to hitherto. It seems that the future of sumanene is very bright and the coming few decades would be the era of buckybowl architectures because a range of interesting properties of these systems are yet to be unmasked. To our perception, the next stage for the chemistry of sumanene is to study the crystals engineering such as the polarity of the crystals, use of sumanene derivatives as catalysts and also to prepare novel sumanene-based electro-active functional materials. Therefore, we believe that still more efficient and practical synthetic methods involving user-friendly chemicals under operationally simple reaction conditions are of pressing need.
